# Prolonged Intracellular Na^+^ Dynamics Govern Electrical Activity in Accessory Olfactory Bulb Mitral Cells

**DOI:** 10.1371/journal.pbio.1002319

**Published:** 2015-12-16

**Authors:** Asaph Zylbertal, Anat Kahan, Yoram Ben-Shaul, Yosef Yarom, Shlomo Wagner

**Affiliations:** 1 Department of Neurobiology, Institute of Life Sciences, Hebrew University and the Edmond and Lily Safra Center for Brain Sciences, Jerusalem, Israel; 2 School of Medicine, Department of Medical Neurobiology, Hebrew University, Jerusalem, Israel; 3 Sagol Department of Neurobiology, University of Haifa, Haifa, Israel; Harvard University, UNITED STATES

## Abstract

Persistent activity has been reported in many brain areas and is hypothesized to mediate working memory and emotional brain states and to rely upon network or biophysical feedback. Here, we demonstrate a novel mechanism by which persistent neuronal activity can be generated without feedback, relying instead on the slow removal of Na^+^ from neurons following bursts of activity. We show that mitral cells in the accessory olfactory bulb (AOB), which plays a major role in mammalian social behavior, may respond to a brief sensory stimulation with persistent firing. By combining electrical recordings, Ca^2+^ and Na^+^ imaging, and realistic computational modeling, we explored the mechanisms underlying the persistent activity in AOB mitral cells. We found that the exceptionally slow inward current that underlies this activity is governed by prolonged dynamics of intracellular Na^+^ ([Na^+^]_i_), which affects neuronal electrical activity via several pathways. Specifically, elevated dendritic [Na^+^]_i_ reverses the Na^+^-Ca^2+^ exchanger activity, thus modifying the [Ca^2+^]_i_ set-point. This process, which relies on ubiquitous membrane mechanisms, is likely to play a role in other neuronal types in various brain regions.

## Abstract 2

An experimental and computational study reveals a novel mechanism for persistent activity of neurons in response to transient stimulation. Instead of involving feedback mechanisms, it relies on slow changes in intracellular sodium ion concentration, leading to prolonged calcium-dependent inward current.

## Author Summary

The accessory olfactory system is essential for chemical communication in animals during social interactions. During this process, the principle cells of the accessory olfactory bulb (AOB) may respond to transient stimulation with prolonged activity, sometimes lasting for minutes—a property known as persistent activity. This property, which has been observed in other brain areas, is usually attributed to positive feedback mechanisms either at the cellular or the network level. Here, we show how persistent activity can emerge without feedback, relying on slow changes in internal ionic concentrations, which keep a record of past neuronal activity for long periods of time. We used a combined computational and experimental approach to show that the complex interaction between various ions, their extrusion mechanisms, and the membrane potential leads to stimulus-dependent persistent activity in the AOB. The same mechanism may apply to other neuronal types in various brain regions.

## Introduction

The accessory olfactory system, also known as the vomeronasal system, mediates chemical communication between conspecifics of most mammalian and reptilian species during social interactions [1]. Inputs to this chemosensory system originate from the sensory neurons of the vomeronasal organ (VNO) that synapse on the mitral cells of the accessory olfactory bulb (AOB), which provide the output of the bulb [2]. Previously, we have shown that AOB mitral cells in vitro respond to brief afferent nerve stimulation with persistent firing activity lasting several minutes [3].

Persistent activity, defined as the ability of neurons to remain active in the absence of external inputs, was documented in many brain areas. Such activity enables the brain to maintain an internal state without continuous external input. It has been suggested that persistent activity is a neuronal correlate of working memory [4], and that it can mediate neuronal integration over long time scales [5].

The time scale of persistent activity (>1 min) is much longer than that of most biophysical mechanisms (typically 0.5–100 ms). Most attempts to explain how the extremely prolonged time scales of persistent activity emerge from such rapid biophysical processes have involved feedback mechanisms [6]. Such feedback can be implemented with recurrent excitation at the network level [7–9], or alternatively, by biochemical pathways at the cellular level. An example of the latter is the mechanism proposed to underlie persistent activity in the entorhinal cortex [10,11] and hippocampal CA1 pyramidal neurons [12,13]. The mechanism involves an interaction between Ca^2+^ influx during spiking and a calcium-activated non-selective (CAN) cation conductance that depolarizes the cell. However, theoretical models of prolonged spiking based on feedback mechanisms are hard to construct in a way that is robust to small parameter changes, immune to noise and continuously graded [10,14–16].

Persistent activity in AOB mitral cells was shown to depend upon Ca^2+^ influx and CAN conductance. However, this intrinsic cellular mechanism does not depend on a feedback cycle involving ongoing neural activity, as persistent firing readily resumes after a temporal firing cessation [3].

In the present study we combined electrophysiological, imaging, and computational approaches to explore the mechanisms underlying persistent firing in AOB mitral cells. We describe a novel mechanism involving interplay between homeostatic processes controlling intracellular Na^+^ and Ca^2+^ concentrations. This novel mechanism, which does not rely upon feedback, is both resistant to noise and allows multiple stable firing states.

## Results

### AOB Mitral Cells Are Capable of Responding to Transient Stimuli with Persistent Firing, Both In Vitro And In Vivo

**Fig 1:**
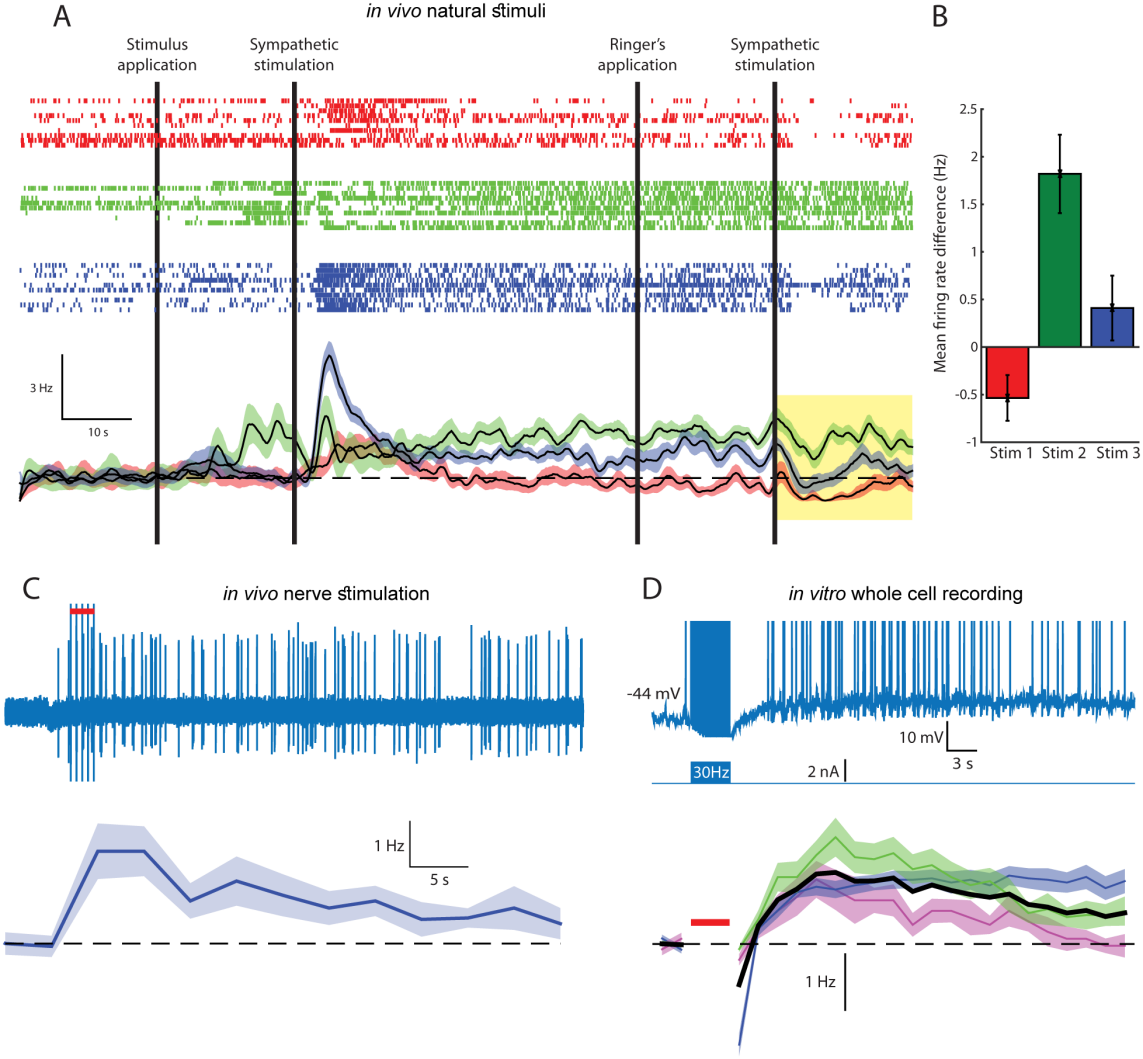
AOB mitral cells are capable of responding with persistent firing to transient stimuli both in vivo and in vitro. (A) Top: raster plots showing a single unit response to ten repetitions of VNO stimulation by female saliva (red), urine (green), and vaginal secretion (blue). Bottom: Peristimulus time histogram (PSTH) of the changes in firing frequency. Vertical black lines denote the times of application of the solutions to the nasal cavity and the activation of the sympathetic nerve. (B) Change in the mean firing frequency, measured during 20 s following stimulus flush (highlighted interval in A) compared with the initial firing rate for the same unit and stimulus. Error bars denote standard error of the mean (SEM). (C) Top: in vivo response of a single unit in the AOB to a brief train of electrical stimuli delivered to the vomeronasal nerve fibers (red bar). Bottom: PSTH showing the mean change in firing frequency over seven repetitions of the same stimulation protocol. (D) Top: the response of an AOB mitral cell (upper panel), recorded in vitro using whole cell patch technique, following a 30 Hz spike train evoked by pulse current injections (lower panel). Spikes are truncated (at −10 mV) to show the underlying plateau potential. Bottom: a PSTH showing the mean change in firing frequency of three cells (ten repetitions in each, same time scale and stimulus as in the top panel). Thick black line: mean PSTH of the three cells. Red bar denotes 4 s of 30 Hz stimulation. Shaded areas denote SEM. See also S1 Fig.

Prolonged firing activity of AOB mitral cells was demonstrated in behaving mice during social investigation of conspecifics [17]. It has remained unclear whether this sustained activity reflects the continuous detection of the stimulus or network properties. In order to explore this issue, we examined AOB responses in anesthetized mice following well-controlled chemosensory stimulus application to the VNO (Fig 1A and 1B, S1 Fig) [18].

While response dynamics often matched those attributed to the vomeronasal pump [18,19], in other cases, elevated firing rates remained high well beyond this time scale, sometimes even after the stimulus was flushed from the nasal cavity and the VNO. Under a highly strict statistical criterion (see Data Analysis in Materials and Methods), reliable cases of persistent activity were found in about one percent (*n* = 7) of the recorded units, and were associated with a particular stimulus, while other stimuli elicited only transient response in the same cells (Fig 1A and 1B). This stimulus selectivity is consistent with a requirement for a high level of activation to trigger the prolonged firing (see below).

Similarly, prolonged single unit AOB spiking activity could be readily elicited in anesthetized mice by direct stimulation of the vomeronasal nerve with a metal electrode (Fig 1C), further confirming that the sustained responses are independent of VNO dynamics. Finally, In agreement with our previous study [3], persistent firing could be elicited in AOB mitral cells in brain slices. An example is shown in Fig 1D (top), where a 4 s train of action potentials is followed by a prolonged period of persistent spiking at a rate of 1–3 Hz lasting for over a minute. The reproducibility of this firing epoch is demonstrated by the mean rate response for the three recorded cells (Fig 1D, bottom). Altogether, these results and the results of our previous studies [3,20] prove that AOB mitral cells are capable of persistent firing responses, both in vitro and in vivo to either electrical or chemical sensory stimulation.

### Persistent Firing Activity in AOB Mitral Cells Involves a Prolonged Depolarizing Current with Complex Dynamics

**Fig 2:**
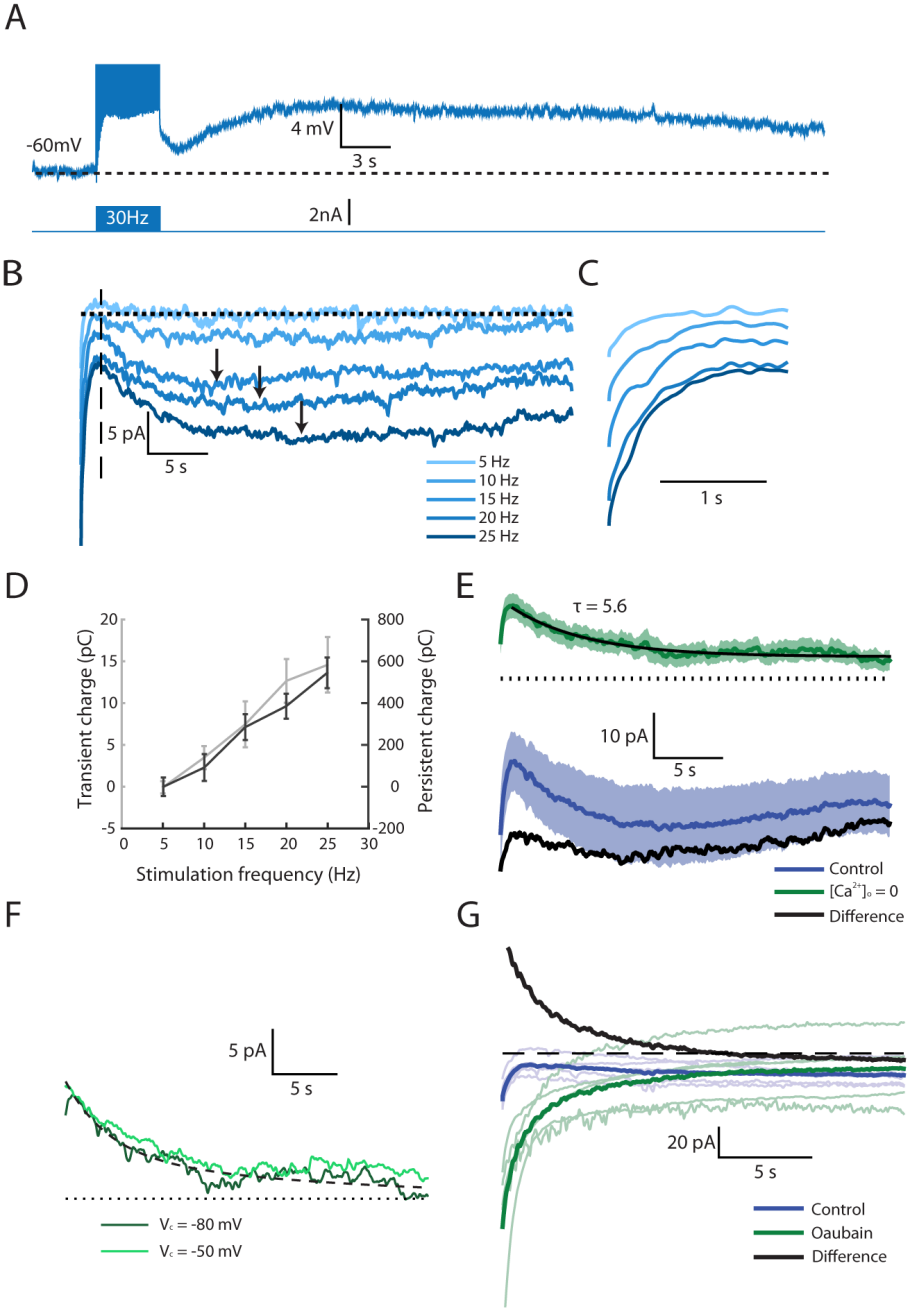
The complex current response following a spike train is comprised of a Na^+^-dependent outward current and a prolonged Ca^2+^-dependent inward current. (A) Mean voltage response (*n* = 4 cells) to a 30 Hz spike train. A hyperpolarizing current was injected throughout the recording to prevent firing before and after the stimulus train. (B) Mean current responses (*n* = 6–10 cells per trace) following a 4 s long spike train at multiple frequencies (denoted by different shades). Arrows point to the peak of the filtered signals. (C) Magnified view of the initial 3 s of the currents shown in (B), revealing the initial transient phase. (D) The charge transferred (current-time integral) as a function of stimulus frequency. Gray curve: the charge transfer between t = 0.25 s and t = 1.5 s post stimulation (transient phase, up to the vertical dashed line in B). Black curve: the charge transferred between t = 1.5 s and t = 42 s post stimulation (persistent phase). Error bars denote SEM. (E) The mean current (*n* = 11 cells) following an evoked 4 s long spike train at 30 Hz before (blue) and after removal of Ca^2+^ from the bath solution (green). The difference between the two traces is shown in black, presumably reflecting the prolonged Ca^2+^-dependent current per se. Shaded areas denote SEM. (F) The outward current recorded after Ca^2+^ removal at a holding voltage of −80 mV (dark green) and −50 mV (light green). (G) The mean inward current (*n* = 5 cells) following an evoked 4 s long spike train at 30 Hz before (blue) and after application of the Na^+^-K^+^ pump blocker ouabain (green). The difference between the two traces is shown in black. Low opacity traces show the average responses of individual cells.

Conducting the in vitro protocol described above while shifting the membrane potential to −60 mV (Fig 2A) blocked the persistent firing and unmasked a prolonged depolarization with a similar time course as the firing activity (compare to Fig 1D). To analyze the currents underlying the prolonged depolarization, the hybrid-clamp methodology was used (see Materials and Methods). Cells were voltage-clamped to −80 mV and trains of action potentials at various frequencies were delivered during a 4 s long current-clamp period. The evoked inward current (Fig 2B) comprised an initial, rapidly decaying phase (*transient phase*, enlarged in Fig 2C), followed by a second, prolonged phase (*persistent phase*), that peaked after >10 s (Fig 2B, arrows) and slowly decayed with a more prolonged time course (>30 s). Notably, the charge transfer during each of the phases monotonically increased with the stimulus frequency (Fig 2D). Thus, the prolonged inward current underlying persistent firing in AOB mitral cells seems to involve transient and persistent components that are proportional to the firing frequency during the stimulation.

### The Prolonged Inward Current Is the Sum of a Na^+^-K^+^ Pump-Mediated Outward Current and a Ca^2+^-Dependent Inward Current (I_CAN_)

The complex dynamics of the prolonged inward current suggest that multiple biophysical mechanisms are involved. To isolate the participating processes, we abolished the inward current, previously shown to be mediated by Ca^2+^-dependent, CAN conductance [3]. Removal of Ca^2+^ from the extracellular solution, as well as blocking the increase in [Ca^2+^]_i_ by adding 5 mM BAPTA to the pipette solution (S2 Fig), abolished the prolonged inward current. Under these conditions, the stimulating train was followed by an outward current of 18±3 pA that monotonically decayed with a single time constant (τ = 5±1 s, *n* = 11 cells, Fig 2E, green trace). Subtracting the outward current from the control condition current (Fig 2E, blue trace) yields a net inward current (Fig 2E, black trace), which is likely due to the CAN conductance. Similar results were previously obtained by blocking N/R type voltage-sensitive Ca^2+^ channels [3].

As shown in Fig 2F, the outward current measured in the absence of Ca^2+^ ions was independent of membrane potential, suggesting that it is not mediated by ionic conductance. The most likely candidate for a voltage-insensitive outward current is an ionic pump current, such as the one produced by the plasma membrane Na^+^-K^+^ pump (Na^+^-K^+^ ATPase) [21]. As shown in Fig 2G (green), blocking the Na^+^-K^+^ pump using ouabain unmasks a strong net inward current peaking immediately after the spike train. The difference between the currents before and after ouabain application is a net outward current resembling the one measured in the absence of Ca^2+^ ions (Fig 2G, black). Thus, the outward current is most likely mediated by the Na^+^-K^+^ pump.

Overall, these data suggest that the complex dynamics of the prolonged inward current reflect the sum of two opposing currents—a voltage-independent outward current (Na^+^-K^+^ pump) decaying over a few seconds and a prolonged Ca^2+^-dependent inward current (I_CAN_) that remains active for minutes.

### The Magnitude of the Inward Current Is Correlated with [Ca^2+^]_i_ at the Dendritic Tuft

**Fig 3:**
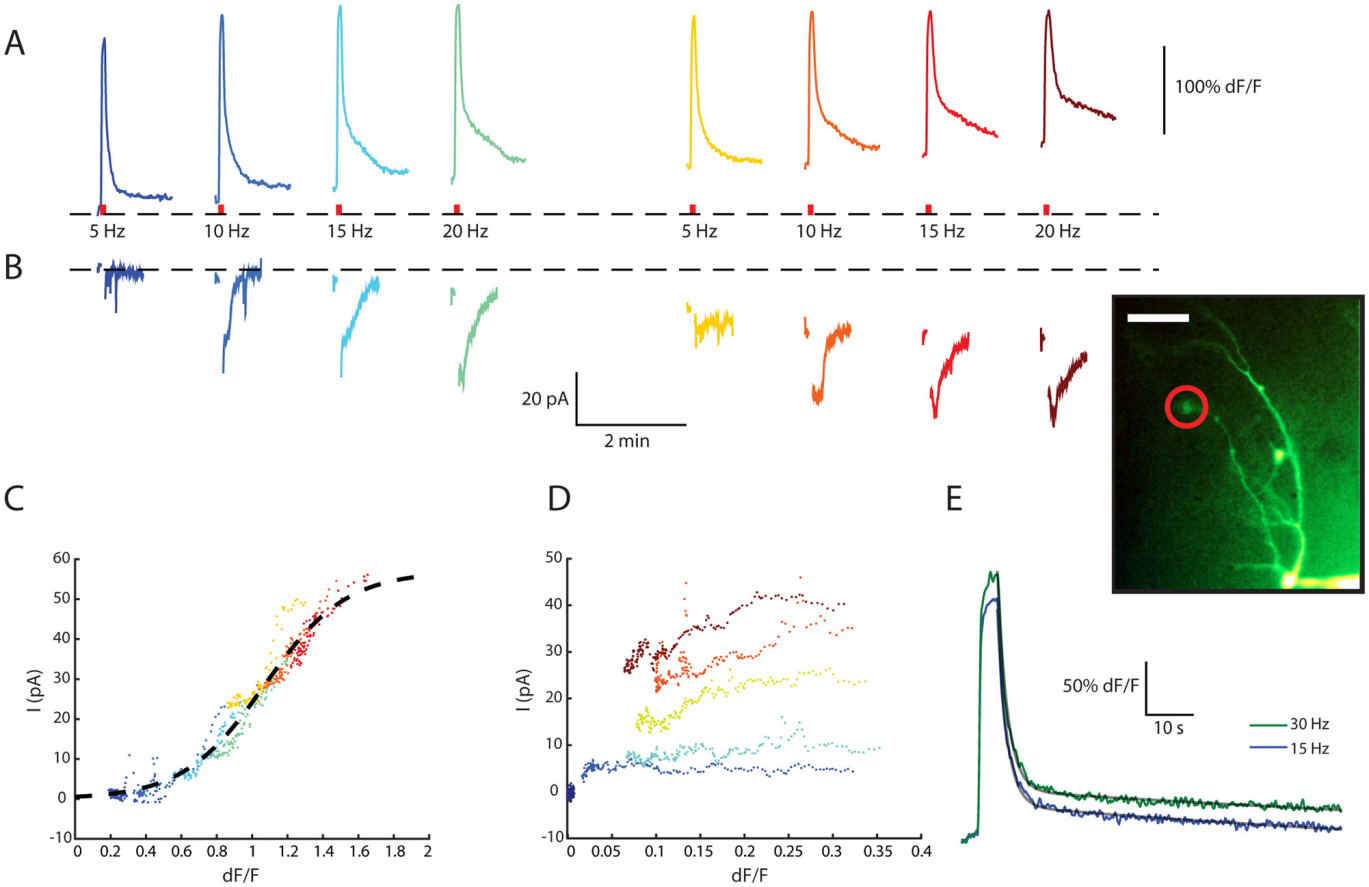
The dynamics of the prolonged inward current is directly related to the Ca^2+^ concentration in the dendritic tuft, enabling stimulus integration over extended time scales. (A) Fluorescence signal recorded from the dendritic tuft during repeated stimulation by spike trains (4 s long at 5, 10, 15, and 20 Hz, red bars). Inset: fluorescence microscope image of the mitral cell filled with OGB-1, showing the dendritic tuft from which the signals were recorded (red circle). Scale bar is 50 μm. The fluorescence level recorded before the first train (dashed line) was used as F_min_ to calculate dF/F values ([F-F_min_] / F_min_). (B) Current traces recorded simultaneously along with the fluorescence signals shown in (A). (C) A scatter plot of the recorded current (B), calculated relative to the current recorded before the first train, versus the simultaneously recorded dendritic tuft fluorescent signal (A), color coded as in (A) and (B). The data of the first 7 s following each spike train were discarded. A sigmoid curve was fitted to the data (dashed line). (D) Same as (C), with somatic fluorescence signals. (E) The mean fluorescence signal in the dendritic tuft during responses to 15 Hz (blue) and 30 Hz spike trains (green). Both signals can be modeled as a sum of two exponential functions (gray lines), one having a short time constant (~2 s) and the other a very long one (~200 s). See also S3 Fig.

To study the spatio-temporal relationship between [Ca^2+^]_i_ and I_CAN_, we correlated [Ca^2+^]_i_ indicator fluorescence in various cellular compartments with the simultaneously recorded somatic inward current. To that end, AOB mitral cells were filled with a Ca^2+^ indicator using the patch pipette (inset in Fig 3). Then, tuft fluorescence (Fig 3A) and the corresponding inward currents (Fig 3B) were simultaneously monitored as trains of action potentials at various frequencies were delivered via the patch pipette to activate the neurons. The transient increase in [Ca^2+^]_i_ in the dendritic tuft was followed by an extremely prolonged decay lasting longer than the interval between stimuli, resulting in summation of [Ca^2+^]_i_ levels over consecutive trains (Fig 3A). Similarly, the prolonged inward current also persisted longer than the interval between stimuli, resulting in progressive increase in inward current as well (Fig 3B).

To analyze the relationship between dendritic [Ca^2+^]_i_ and the inward current, the current amplitude at each time point was plotted against the simultaneously measured fluorescence level. Fig 3C shows this analysis, applied to the data shown in Fig 3A and 3B. As apparent, the inward current shows a clear sigmoidal dependence on the fluorescence signal, suggesting that the [Ca^2+^]_i_ in the dendritic tuft tightly correlates with the slow dynamics of inward current (see S3E and S3F Fig for more examples). In contrast to tuft fluorescence, somatic fluorescence does not correlate with the magnitude of the inward current (Fig 3D, S3A and S3D Fig). The close relationship between tuft [Ca^2+^]_i_ and the magnitude of the inward current suggests that the prolonged current reflects the extended elevation of tuft [Ca^2+^]_i_. Indeed, close examination of tuft fluorescence levels (Fig 3E) revealed that the decay of [Ca^2+^]_i_ in the tuft followed two distinct time scales: fast initial decay (τ = 1.9 s, mean of three cells) followed by very slow decay (τ = 47.0 s). This slow process suggests that [Ca^2+^]_i_ is in a quasi-stable state, the level of which is determined by the stimulus frequency. Consistent with this, increasing the stimulation frequency from 15 Hz to 30 Hz almost doubled the quasi-stable state level (Fig 3E, blue and green traces).

Overall, these results suggest that the inward current underlying persistent firing of AOB mitral cells is mediated by dendritic Ca^2+^-dependent ionic conductance (CAN) and that its slow dynamics likely reflect a complex interaction between several ionic extrusion mechanisms.

### A Hypothetical Mechanism Involving the Na^+^-K^+^ Pump, the Plasma Membrane Ca^2+^ Pump, and the Na^+^-Ca^2+^ Exchanger Can Explain the Observed Slow Current Dynamics

**Fig 4:**
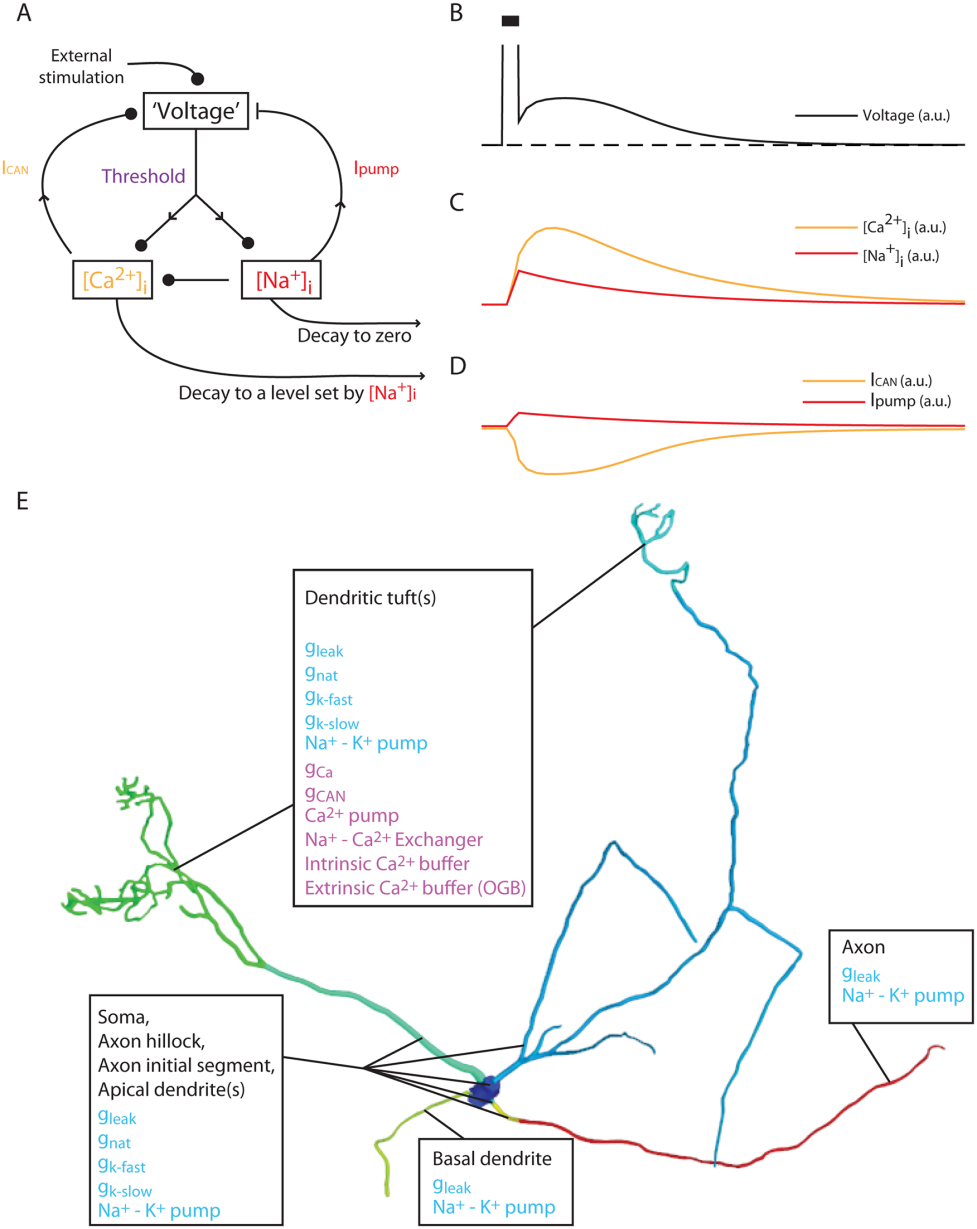
A simple abstract model, based on the interactions between ionic extrusion mechanisms, reproduces the long-term voltage behavior observed in mitral cells. (A) A schematic description of the abstract model—the “voltage” is the sum of an external stimulation, inward [Ca^2+^]_i_-dependent current (I_CAN_) and outward [Na^+^]_i_- dependent current (I_pump_). [Ca^2+^]_i_ and [Na^+^]_i_ influx rates are determined linearly by the “voltage” once it crosses a threshold. [Na^+^]_i_ exponentially decays to zero, while [Ca^2+^]_i_ decays to a level set by [Na^+^]_i_. I_CAN_ and [Ca^2+^]_i_ have a sigmoidal relationship, while I_pump_ and [Na^+^]_i_ have a logarithmic one. (B)–(D), the dynamics of: (B) “voltage;” (C) [Ca^2+^]_i_ (orange) and [Na^+^]_i_ (red); (D) I_CAN_ (orange) and I_pump_ (red) when running the model with transient external stimulation (black bar). All time and quantity units are arbitrary. (E) A description of the detailed conductance-based model, showing the compartmental distribution of the channels, pumps, and exchangers overlaid on the reconstruction of the mitral cell used for the model. g_leak_: leak conductance, composed from separate passive Na^+^ and K^+^ conductances; g_nat_: transient voltage-gated Na^+^ conductance; g_k-fast_/g_k-slow_: fast and slow voltage-gated K^+^ channels; g_Ca_: voltage-gated Ca^2+^ conductance; g_CAN_: Ca^2+^-dependent non-specific cation conductance.

The result described above, in which the tuft [Ca^2+^]_i_ decays to a quasi-stable state determined by the stimulation frequency, suggests that the quasi-stable state is generated by slowly changing, activity-dependent quantity. One such quantity may be the tuft [Na^+^]_i_, which affects the Ca^2+^ dynamics by interacting with ionic transport mechanisms such as the Na^+^-Ca^2+^ exchanger. This exchanger, which is the major mechanism for control of large excess Ca^2+^ [22], uses the Na^+^ electrochemical gradient to extrude Ca^2+^. Thus, increase in [Na^+^]_i_ which leads to a decreased Na^+^ gradient, reduce or even reverse the Ca^2+^ flux through the exchanger [23]. We examined this possibility in a simple abstract dynamical model, with a minimal number of parameters (Fig 4A; see S1 Text for a description of the model equations). In this model, [Ca^2+^]_i_ and [Na^+^]_i_ increase at a rate proportional to an abstract “voltage” quantity, given that the “voltage” is above a certain threshold. [Na^+^]_i_ decays exponentially to zero over time, while [Ca^2+^]_i_ decays to a level linearly determined by [Na^+^]_i_ (the quasi-stable state). The “voltage” is a sum of three components: externally applied current, inward Ca^2+^-dependent current, and outward (negative) Na^+^-dependent “pump” current.

Fig 4B and 4D shows the results of running this model with a pulse of externally applied current (black bar). As apparent, the “voltage” (Fig 4B) behavior qualitatively resembles the experimental observations (compare to Figs 1D and 2A). This voltage trajectory is due to the changes in [Na^+^]_i_ and [Ca^2+^]_i_ (Fig 4C) and the corresponding currents (Fig 4D). Thus, the feasibility of the mechanism suggested above is confirmed by this simple abstract model. In order to further test this hypothetical mechanism and produce quantitative predictions, we incorporated the principles of this mechanism into a realistic conductance-based model.

### A Conductance-Based Model Reproduces the Dynamics of the Ca^2+^-Dependent Fluorescence and the Membrane Currents

A realistic conductance-based model (see Materials and Methods), was constructed using the detailed morphology of a single typical mitral cell (Fig 4E and S4 Fig) for which the electrophysiological properties were characterized. The model assumes that active conductances reside in the apical dendrites and dendritic tufts, as well as in the soma and axon initial segment [24], so that [Na^+^]_i_ increase in these compartments following firing. A novel feature of our model is the incorporation of compartmental [Na^+^]_i_ as state variables along with longitudinal ionic diffusion. Accordingly, [Na^+^]_i_ not only sets the local Na^+^ reversal potential but also affects localized ionic extrusion mechanisms (Na^+^-K^+^ pumps, Na^+^-Ca^2+^ exchangers). The Ca^2+^ influx, buffering and extrusion mechanisms (including a simulated Ca^2+^ indicator), as well as a Ca^2+^ dependent conductance, were introduced in the dendritic tufts. The spatial distribution of membranal mechanisms in the model is shown in Fig 4E (See Materials and Methods for a link to the model source code and S1 Text for a full description of the model equations and parameters). Using such a model, one can calculate the temporal dynamics of [Na^+^]_i_ and [Ca^2+^]_I_ in various cellular compartments.

**Fig 5:**
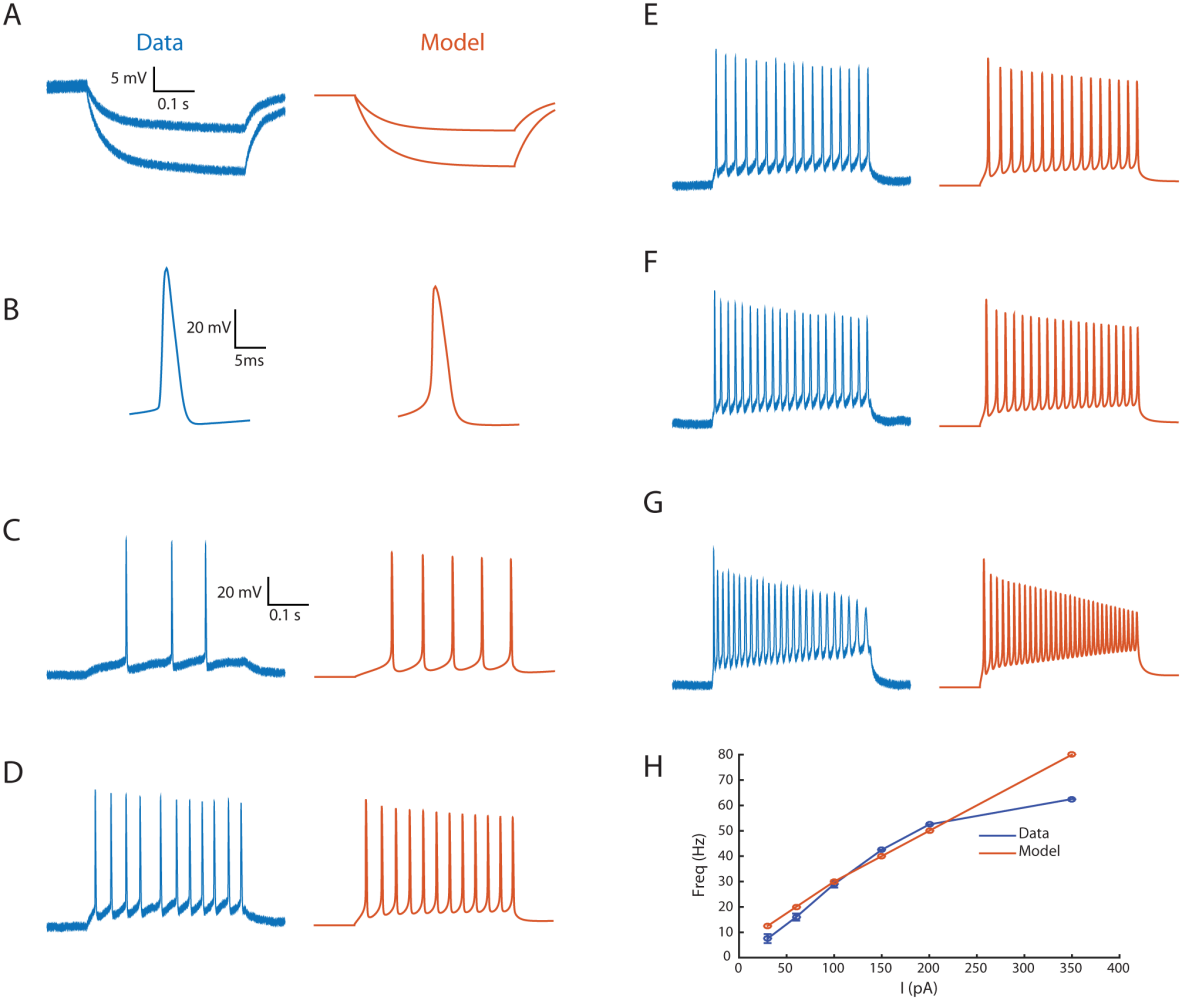
The passive and firing properties of an AOB mitral cell are reproduced by a conductance-based model of a reconstructed cell. (A)–(G) Comparison between the experimental observation (blue) and the model prediction (orange) of the: (A) Mean voltage response to a hyperpolarizing current steps (30 and 60 pA). (B) Mean spike trajectory. (C)–(G) Firing responses to step current injections of 30, 100, 150, 200, and 350 pA, respectively. (H) I-f curve of the real (blue) and model (orange) cells. See also S4 and S5 Figs.

An evolutionary multi-objective algorithm [25,26] was used to find the biophysical parameters that best fit our electrophysiological observations. An initial evolutionary process was employed to find the best fit to the following measured parameters: the response to a hyperpolarizing current pulse (Fig 5A), the shape of the action potential (Fig 5B), the modulation of the spike amplitude during a strong (350 pA) current injection (Fig 5G), and the I-f curve (Fig 5H). As shown, the model accurately reproduces the behavior of the real cell with respect to these objectives. Notably, the spike amplitude modulation during depolarizing current injection simulated by the model precisely fitted the experimental observations, despite the fact that only a 350 pA current injection was used as an objective (Fig 5E–5G). Importantly, the spike amplitude modulation in the model was the result of [Na^+^]_i_ accumulation during the spike train and would not be reproduced when [Na^+^]_i_ accumulation was prevented (S5 Fig).

**Fig 6:**
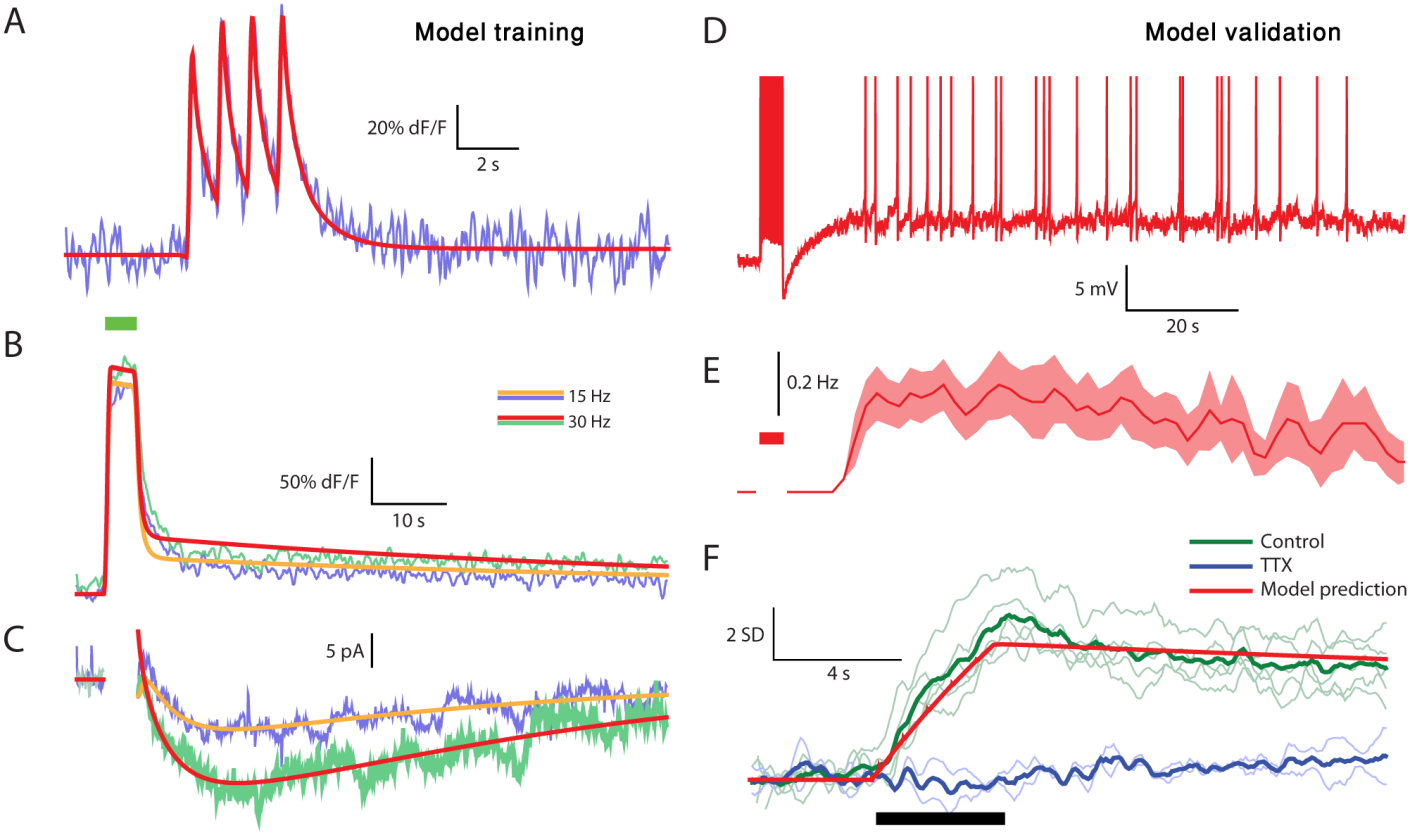
The slow tuft fluorescence signal and the prolonged inward current are reproduced by a conductance-based model of a reconstructed mitral cell, which is validated by producing persistent firing and predicting Na^+^ imaging results. (A) Tuft fluorescence signal during a train of four spikes evoked at 1 Hz in the real (light blue) and model (red) cells. (B) Tuft fluorescence signal during and after a 4 s long spike train (green bar) evoked at 15 Hz (blue) and 30 Hz (green) compared to the corresponding simulation results (orange and red lines, respectively). (C) Same as (B), for the current following the stimulation. (D) The model voltage response to a 30 Hz spike train evoked by pulse current injections. Gaussian white noise (σ = 20 pA) was injected during the simulation. (E) A PSTH showing the mean firing frequency in the model cell of ten trials such as the one shown in (D). Shaded area denotes SEM. (F) The Z score (based on pre-stimulus standard deviation) of the Na^+^ indicator SBFI fluorescence signal in the apical dendrite of five cells (ten repetitions in each) in control conditions (green lines) and two cells in the presence of TTX (light blue lines) while a 30Hz train stimulus was applied (black bar). Upward direction denotes decreased fluorescence. Thick lines denote multi-cell average. The red line denotes the tuft [Na^+^]_i_ predicted by the model, scaled in the Y direction. See also S7 Fig.

The goal of the next evolutionary process was to find the parameters that reproduce the tuft Ca^2+^ indicator fluorescence and the prolonged somatic inward current (see Materials and Methods). Trains of action potentials with frequencies of 1, 15, and 30 Hz were used to activate the model neuron. The simulated dendritic tuft fluorescence and the accompanying prolonged inward current were then compared to the experimental observations. At a frequency of 1 Hz (Fig 6A), the simulation (red line) perfectly reproduced the observed fluorescence signal (blue line). At higher frequencies (15 Hz and 30 Hz, Fig 6B), both measured (blue and green lines) and simulated (orange and red lines) fluorescence levels, rapidly increased to saturation levels during the stimulation (Fig 6B, top green bar). The rapid increase was followed by an equally rapid decline to a low quasi-stable level that strongly depended on the stimulation frequency (Fig 6B). Moreover, the simulated prolonged inward current also closely fit the experimentally measured current (Fig 6C).

In order to assess the sensitivity of the model to changes in its parameters, we created a population of 1,200 model neurons. In each model, each parameter (except the channels' half-activation voltage parameters) was randomly selected from a uniform distribution that spanned between -10% and +10% relative to the original value. We then examined, in each of the models, the predicted prolonged inward currents evoked by 30Hz spike train. The properties of the resulting currents distribute normally (see example histograms for the maximum current in S6A Fig and the residual current after 1 min for the train end in S6B Fig). As apparent from the 80% bounds of the distribution (S6C Fig), this change in parameters did not cause a large deviation from the fit of the model to the experimental data.

A critical validation of the model is its ability to reproduce the persistent firing recorded in AOB mitral cells. Indeed, a train of simulated spikes evoked long lasting persistent activity (Fig 6D) which resembled the experimental observations (Fig 1D). Adding Gaussian current noise introduced variability to the responses that upon averaging reproduced the PSTH observed in vitro (compare Figs 6E to 1D).

Another critical validation is the ability of the model to predict the time course of dendritic [Na^+^]_i_, and particularly its rise during stimulation and very slow subsequent decay that maintains a quasi-stable state for the tuft [Ca^2+^]_i_. To that end, we used two fluorescent Na^+^ indicators, sodium-binding benzofuran isophthalate (SBFI) and Sodium Green, to image the dendritic Na^+^ dynamics following a stimulus train [27]. As shown in Fig 6F, the averaged observed dynamics (thick green line) indeed match the predicted dynamics (red line) (For dF/F signal not normalized by standard deviation and similar results using the Sodium Green indicator, see S7 Fig) As shown in the presence of tetrodotoxin (TTX,blue lines), the signal does relate to opening of voltage-gated Na^+^ channels. Notably, similar dynamics were previously observed experimentally in cortical pyramidal neurons [27].

Thus, we conclude that our mitral cell model adequately reproduces the experimental observations.

### The Quasi-stable State of [Ca^2+^]_i_ in the Dendritic Tuft Reflects the Opposing Actions of the Ca^2+^ Pump and the Na^+^-Ca^2+^ Exchanger

**Fig 7:**
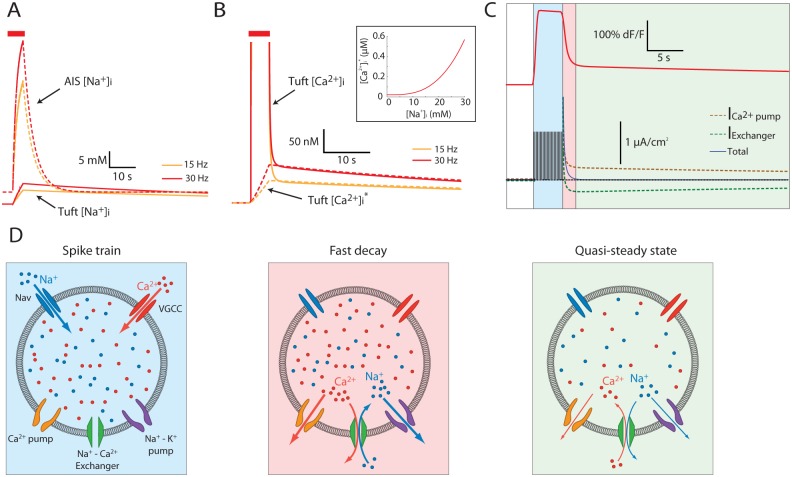
In the model, the prolonged elevation in tuft Ca^2+^ is due to a new quasi-stable state of Ca^2+^, dictated by the tuft [Na^+^]_i_. (A) [Na^+^]_i_ in the axon initial segment (dashed lines) and the dendritic tuft (solid lines) during and after 15 Hz (orange) and 30 Hz (red) stimulations. (B) The tuft [Ca^2+^]_i_ (solid lines, truncated) during and after 15 Hz (orange) and 30 Hz (red) stimulations, along with the quasi-stable state [Ca^2+^]_i_ (dashed lines) calculated according to the relationship between [Na^+^]_i_ and the quasi-stable state [Ca^2+^]_i_ (inset, inferred by running the simulation to relaxation at a range of fixed [Na^+^]_i_). (C) The simulated Ca^2+^ indicator fluorescence (top) and the Ca^2+^ currents (bottom) during and after a 30 Hz stimulation. The Na^+^-Ca^2+^ exchanger Ca^2+^ current (green) and the Ca^2+^ pump current (orange) are summed to almost zero (blue) soon after the stimulus, creating a new quasi-stable state. (D) Schematic representations of a section in the dendritic tuft, showing the model mechanisms dictating [Na^+^]_i_ and [Ca^2+^]_i_ during the spike train (left), during the initial decay when both the exchanger and the pump extrude Ca^2+^ (middle), and during the quasi-stable state when the two processes oppose each other (right). Background colors correspond to the colors in (C).

We used this model to examine possible mechanisms underlying the prolonged current responses of AOB mitral cells. We first examined the time course of [Na^+^]_i_ in two cellular compartments: the axon initial segment (AIS) and the dendritic tuft, while the model neuron was activated by a 4 s train of 15 or 30 Hz (Fig 7A, orange and red traces, respectively). The model predicts that during the stimulus, [Na^+^]_i_ at the AIS (dashed lines) would reach a very high level (50 and 63 mM for 15 and 30 Hz stimulation, respectively), followed by a relatively rapid decline (τ = 3.85 s) to baseline levels. The large increase in AIS [Na^+^]_i_ is due to the high density of voltage-gated Na^+^ channels and the limited volume of the compartment. The relatively fast recovery results from the activity of the Na^+^-K^+^ pump and the fast diffusion to the compartments adjacent to the AIS (axon and soma) that serve as diffusion sinks.

In contrast to the AIS, tuft [Na^+^]_i_ increased only to a moderate level (15 and 17 mM for 15 and 30 Hz stimulation—solid orange and red traces in Fig 7A, respectively), followed by extremely slow exponential recovery over a time course of minutes (τ = 130 s and 115 s for 15 and 30 Hz stimulation, respectively). This slow time course, also observed in real mitral cells using Na^+^ fluorescent indicators (Fig 6F), stems from the slow diffusion in the thin dendritic process and the low density of Na^+^-K^+^ pumps in this compartment.

The prolonged elevated [Na^+^]_i_ in the dendritic tuft is bound to affect the Na^+^-Ca^2+^ exchanger, hence to determine the quasi-stable state of [Ca^2+^]_i_. We examined this prediction using the model by calculating the stable-state [Ca^2+^]_i_ for various fixed [Na^+^]_i._ values. As shown in Fig 7B (inset), an increase in [Na^+^]_i_ elevated the stable-state [Ca^2+^]_i_ non-linearly. This relationship was used to calculate the quasi-stable state of [Ca^2+^]_i_ during and after the stimulation train, based on the instantaneous values of [Na^+^]_i_. As shown in Fig 7B for stimulation frequencies of 15 Hz and 30 Hz, the simulated tuft [Ca^2+^]_i_ (solid lines) quickly dropped to its quasi-stable state level (dashed lines), and then closely followed the slow decrease of the quasi-stable state. The proposed mechanism that maintains [Ca^2+^]_i_ in a quasi-stable state level is demonstrated in Fig 7C and 7D, where the Ca^2+^ currents of the exchanger (dashed green line) and the Ca^2+^ pump (dashed orange line) are shown along with schematic diagrams depicting each state. Immediately following the stimulus train, both currents are positive (outward, Fig 7D, middle), but then the exchanger current becomes negative (inward) while the pump current remains positive. As a result, the net current of the Ca^2+^ regulatory mechanisms reaches a near-zero value (Fig 7C, blue line; Fig 7D, right). The inward current mediated by the exchanger reflects the condition of high [Na^+^]_i_ level, that causes the exchanger to operate in a "reverse mode" (Ca^2+^ influx). In this state [Ca^2+^]_i_ is determined by the slow change of the quasi-stable state, which is due to the slow return of tuft [Na^+^]_i_ back to baseline levels (Fig 7A).

### Blocking the Exchanger by Substituting Na^+^ with Li^+^ Shortens the Duration of Tuft Ca^2+^ Elevation and the Inward Current

**Fig 8:**
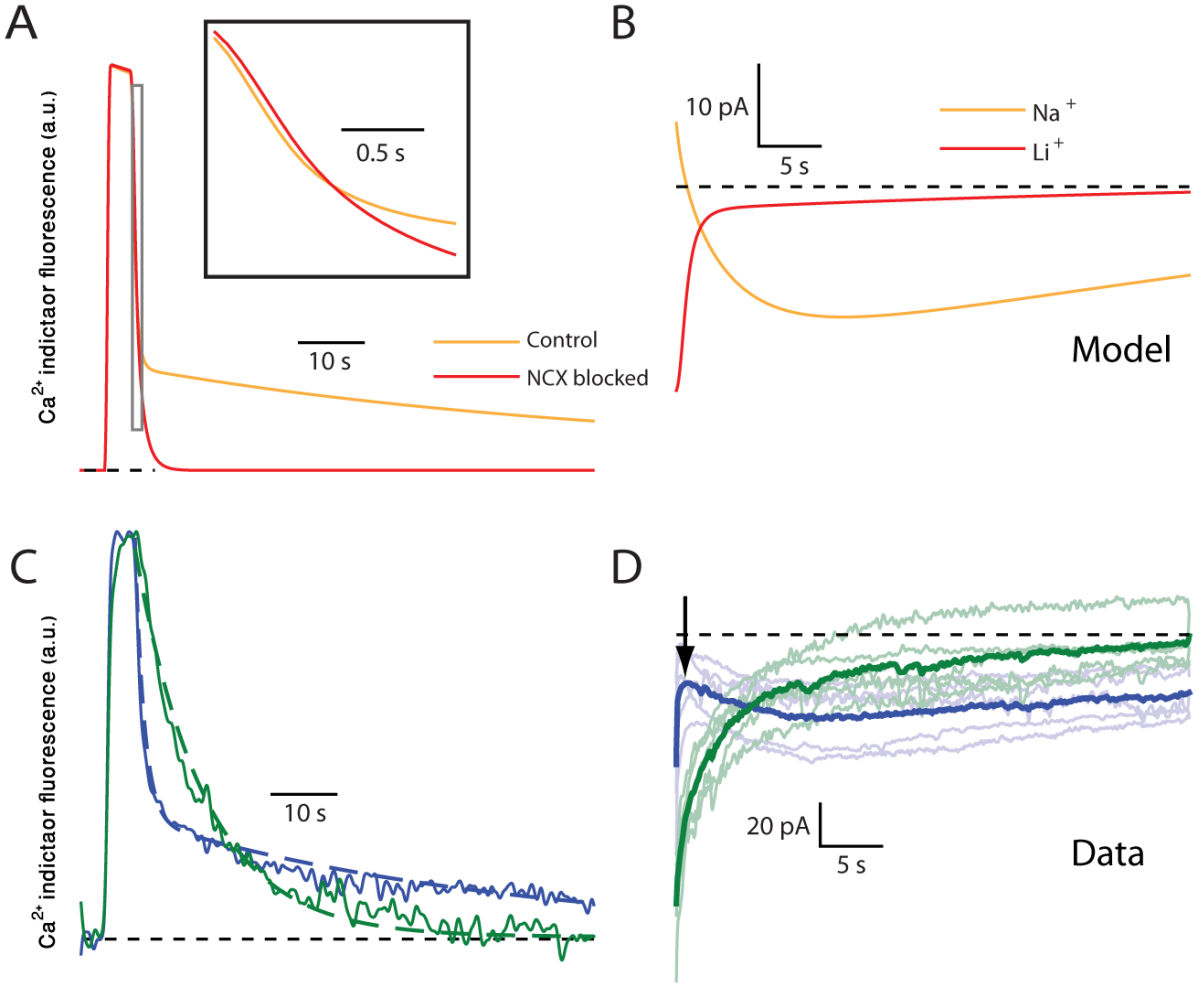
Eliminating the Na^+^-Ca^2+^ exchanger activity by substituting Na^+^ with Li^+^ abolishes the prolonged elevation of tuft [Ca^2+^]_i_ and, hence, the prolonged I_CAN_. (A) The model prediction for the normalized tuft fluorescence following a 30 Hz stimulation under control conditions (orange) and when the Na^+^-Ca^2+^ exchanger is blocked (red). Inset: magnified view of the initial 1 s of decay. (B) The model prediction for the current measured following a 30 Hz stimulation under control conditions (orange) and when the Na^+^-K^+^ pump is fully blocked and the Na^+^-Ca^2+^ exchanger is 60% blocked (simulating the Na^+^-Li^+^ substitution, red). (C) Normalized mean tuft fluorescence signal in a single cell during and after 30 Hz stimulation in normal bath solution (blue) and after substituting Na^+^ with Li^+^ (green). (D) The mean net inward current following an evoked 4 s long spike train at 30 Hz under control conditions (blue) and after substituting Na^+^ with Li^+^ (green). Low opacity traces showing average responses of individual cells (*n* = 5). The abbreviation “a.u.” stands for arbitrary units.

As described above, the prolonged quasi-stable state of [Ca^2+^]_i_ is the result of the opposing actions of the pump Ca^2+^ efflux and Ca^2+^ influx due to the reverse-mode of the Na^+^-Ca^2+^ exchanger (Fig 7C and 7D). Therefore, blockade of the exchanger should result in acceleration of the recovery of [Ca^2+^]_i_. We examined this prediction using the model by calculating the decay of the tuft fluorescence under control conditions (Fig 8A, orange line) and after blocking the exchanger (Fig 8A, red line). Indeed, a much faster return to baseline levels was obtained in the absence of exchanger activity. Experimentally, such blockade can be realized by substituting Na^+^ ions in the extracellular solution with Li^+^, which cannot be transported by the exchanger [28–30]. This manipulation was simulated by modeling the effect of Li^+^ on the exchanger and the Na^+^-K^+^ pump [31,32]. Fig 8B shows that following substitution of Na^+^ by Li^+^ in the model, the slow inward current (orange line) is replaced by a fast, transient inward current (red line) that rapidly declines to baseline.

Similar results were obtained experimentally (Fig 8C and 8D) by substituting Na^+^ with Li^+^ in the bath solution. In the presence of Li^+^ (green) the decay of the fluorescence signal in the tuft (Fig 8C) followed a single time constant (τ = 11 s, dashed line, compare to inset in Fig 8A). Thus, in the absence of exchanger activity the elevated quasi-stable state was blocked. In accordance with the fluorescence measurements, the inward current (Fig 8D) decayed faster in the presence of Li^+^ (compare green to blue lines) and the initial “bump” (arrow in Fig 8D) created by the Na^+^-K^+^ pump outward current was absent (compare to the effect of ouabain application, Fig 2G). These experimental observations strongly support the proposed model in which a quasi-stable state of [Ca^2+^]_i_ is generated by the reversed action of the Na^+^-Ca^2+^ exchanger.

### Simulated Synaptic Input Reproduces a Transition from Transient to Prolonged Firing Response

**Fig 9:**
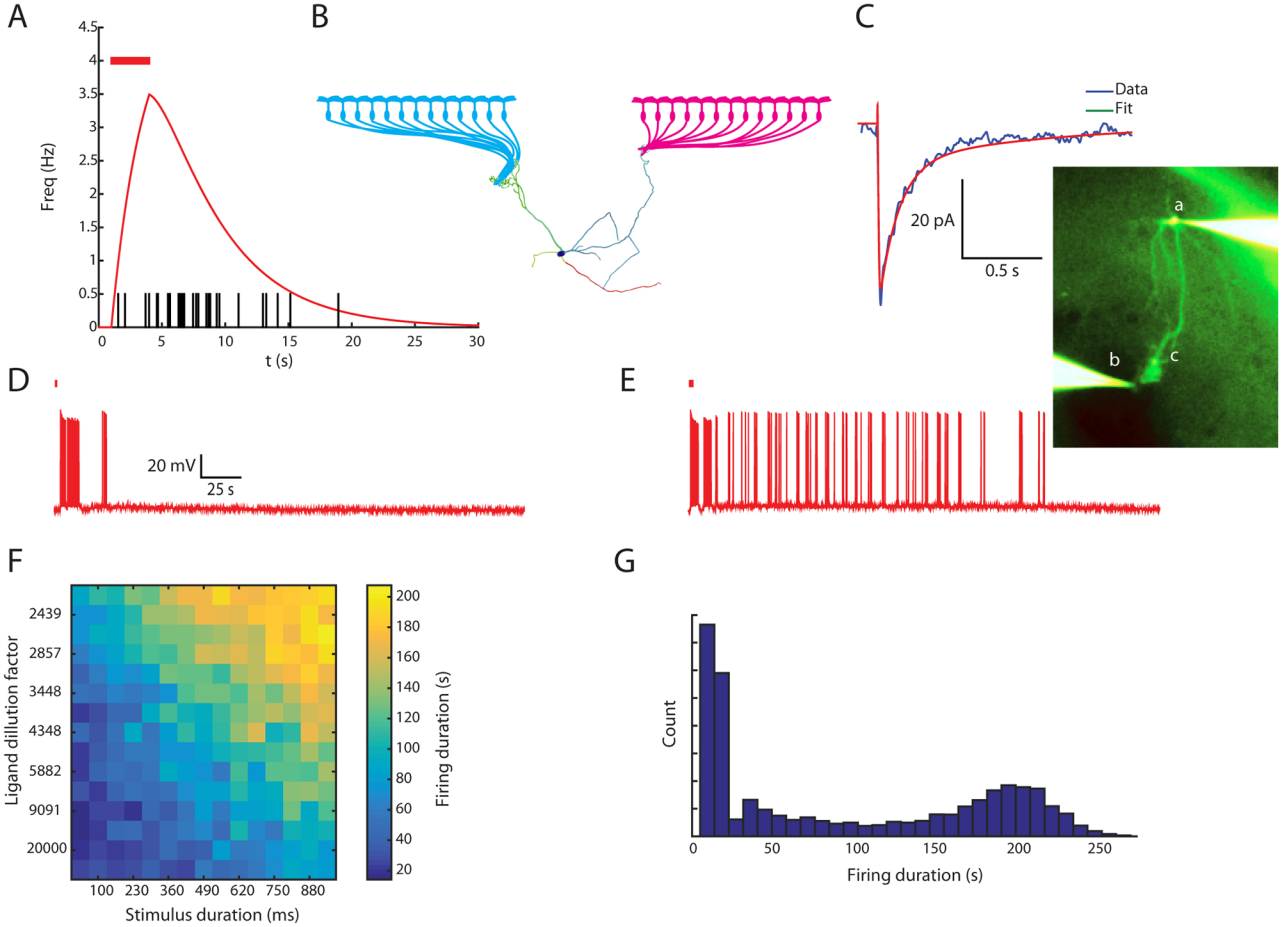
Transitions from transient to persistent firing are predicted by simulating the inputs of a mitral cell during natural stimuli. (A) An example of predicted firing frequency in a VNO sensory neuron (red) based on ligand-receptor interaction during a brief stimulus presentation (red bar), and a corresponding random spike time series (black). (B) Schematic representation of local network simulation, incorporating the VNO sensory inputs to a single mitral cell. (C) Unitary excitatory post-synaptic current (EPSC,blue) recorded at mitral cell soma (a in inset) following stimulation by a bipolar theta electrode (b in inset) located close to the dendritic tuft (c in inset). The synaptic current was represented by double-exponential approximation (red). (D) A transient response of the simulated cell to a weak (short duration and high dilution) stimulus, that was insufficient to trigger persistent firing. (E) A persistent response of the simulated cell to a strong (long duration and low dilution) stimulus. (F) Mean duration of simulated firing in a range of ligand dilution factors (*y*-axis) and stimulus durations (*x*-axis). (G) Relative frequencies of different firing durations on multiple runs with the stimulus concentration and duration ranges as in (F).

We examined the response of our detailed model to VNO inputs using a simple feed-forward network simulation (see Materials and Methods). The input stage of the network represents the responses of vomeronasal sensory neurons (VSNs) to natural stimuli, which follow a simple ligand-receptor interaction [33,34]. A simple model of VSN firing was established by first calculating the predicted time course (Fig 9A, red line) of a response to a brief stimulus application (red bar), and then using it to generate a random spike train (vertical lines). For the purpose of the model, we assumed that each tuft is innervated by 13 VSNs (Fig 9B; within the lower range of reported convergence [35]).

The unitary synaptic response of an AOB mitral cell to sensory fiber stimulation (Fig 9C, blue line) was measured by stimulating the dendritic tuft of a mitral cell (see Materials and Methods, micrograph in Fig 9C). A model of synaptic conductance was fitted to the averaged response (Fig 9C, orange line) and assigned to the dendritic tufts of the aforementioned mitral cell model. The simulated response was then tested for two ligands presented simultaneously to two groups of 13 VSNs, converging on two different dendritic tufts of the model mitral cell (Fig 9B). This simulation was run using a range of stimulus durations for both ligands, and a range of concentrations for one of them (the concentration of the second ligand was kept constant). Random white noise was also injected to the model mitral cell. Using these parameters, we encountered two possible outcomes: one was a transient firing response (Fig 9D) while in the other firing persisted for ~200 s (Fig 9E).

Fig 9F summarizes the average firing durations for different combinations of stimulus duration and concentration. As shown, longer stimulus durations and higher concentrations led to persistent firing responses. The bimodal distribution of the response duration is shown in Fig 9G. As apparent, the response was either transient, following the stimulus application, or persistent peaking at 200 s, as previously demonstrated for AOB mitral cells [3]. Thus, our model cell embedded in a realistic small network simulation reproduces the transition of the responses of AOB mitral cells between transient and persistent modes as a function of stimulation strength and duration, as observed both in vitro and in vivo.

## Discussion

Here, we combined in vivo and in vitro electrophysiological recordings, Ca^2+^ and Na^+^ imaging, and computational modeling to investigate how persistent firing responses are generated in AOB mitral cells. We first demonstrated that these cells are capable of responding in vivo with persistent firing to natural stimuli applied to the VNO, in a stimulus-specific manner. We then used AOB slices to explore the ohmic and non-ohmic currents that underlie the persistent firing responses. The dynamics and inter-relationships of these current sources were analyzed by pharmacological and ionic manipulations. By combining patch-clamp recordings with Ca^2+^ and Na^+^ imaging we showed that the dynamics of the currents underlying persistent firing rely on slow changes in internal ionic concentrations within specific cellular compartments. Using first abstract and then realistic computational models of AOB mitral cell we demonstrated that these changes are governed by passive diffusion, as well as by local active processes involving ionic pumps and exchangers. The model results accurately predict the persistent firing responses, the dynamics of Na^+^ in the dendrites and the results of substituting Na^+^ by Li^+^. Finally, we employed our model in a local network simulation and established that the model cell indeed shifts between transient and persistent firing responses to stimuli detected by VNO neurons, as a function of the duration and strength of stimulation.

### Functional Significance of Persistent Activity in AOB Mitral Cells

We have previously hypothesized [3] that the accessory olfactory system reports the social context to an animal by inducing specific brain states based on the persistent activity of its mitral cells. These states can then change the processing of sensory information in other brain areas [36–38]. This hypothesis is supported by the current study where we showed, both in vivo and in vitro that AOB mitral cells shift from transient to persistent firing responses to stimuli arriving from the VNO. In agreement with our previous reports [3,20], this transition is relatively sharp and depends on sufficient stimulus strength and duration. This may correspond, for example, to the presence of a rich source of social chemosensory cues, e.g., a conspecific animal, which would elicit persistent firing in AOB mitral cells, thereby conveying to higher brain centers the presence of a social partner.

### A Novel Mechanism for Persistent Activity

Persistent activity has been reported in several brain areas, including mitral cells of the main olfactory bulb in vivo [39], and was suggested to mediate working memory or prolonged brain states [4]. To date, most of the mechanisms purposed for persistent activity include either network or biophysical feedback loops [5,10,16,39,40]. We propose a novel mechanism for persistent neuronal activity in AOB mitral cells, in which a train of action potentials that back-propagate to the dendritic tuft [24] elevates the tuft [Na^+^]_i_. This increase in the tuft [Na^+^]_i_ shifts the [Ca^2+^]_i_ stable state upwards [29], thus creating an elevated quasi-stable state for [Ca^2+^]_i_. The slow decay of [Ca^2+^]_i_ is dictated by the slow removal of Na^+^ ions from the tuft (Fig 7). The novelty of our model is in the absence of biochemical, biophysical, or network feedback mechanisms or hysteresis.

### Exploring Alternative Mitral Cell Models

Two alternative models were examined in an attempt to reproduce the observed experimental results. One model incorporated two distinct CAN conductances—a fast one and a slow one (compare with [41]), without activity-dependent outward current. Each of these currents produces a different phase of the observed inward current (Fig 2B and 2C). While this model reproduces the results used as objectives in the model training phase (Figs 5 and 6A–6C), it failed to produce the persistent activity (Fig 6D and 6E), emphasizing the importance of this validation. The long time constant kept the slow Ca^2+^-dependent current away from its stable-state value in the hybrid clamp simulations, but this current would greatly intensify and cause a runaway effect in a current clamp simulation (S8A Fig).

In another model, a single Ca^2+^-dependent non-specific cation conductance was used, along with a Ca^2+^-dependent K^+^ conductance (SK/BK) that account for the outward current observed immediately after the stimulating train of action potentials. Although this model reproduces the results used as objectives in the model training phase (Figs 5 and 6A–6C), it failed to explain the outward current recorded in the absence of [Ca^2+^]_o_ (Fig 2E).

The ultimate rejection of these models demonstrates the importance of the model validation step.

### The Role of Na^+^ As an Integrator of Spiking Activity

According to our results, [Na^+^]_i_ plays a key role in both transient and prolonged biophysical processes. First, increase in AIS [Na^+^]_i_ alters the Na^+^ Nernst potential, thus lowering the spike amplitude (Fig 5E–5I and S5 Fig). Second, elevation in [Na^+^]_i_ increases the Na^+^-K^+^ pump-mediated outward current that terminates bursts of activity by hyperpolarizing the cell (Fig 2E). Third, increase in [Na^+^]_i_ decreases Ca^2+^ efflux by modulating the Na^+^-Ca^2+^ exchanger (Fig 8). The latter endows [Na^+^]_I_ with the ability to dictate [Ca^2+^]_i_ and thus the inward I_CAN_.

In thin and slightly active processes like the dendritic tuft, [Na^+^]_i_ is only moderately increased by neuronal activity and its extrusion is exceptionally slow (Fig 6F,[27]). Thus, [Na^+^]_i_ can attain a range of quasi-stable states—a property that renders it an ideal candidate to integrate epochs of high neural activity. This is in contrast to [Ca^2+^]_i_, which is highly dynamic as a function of neuronal activity on one hand, and rapid extrusion on the other.

Notably, changes in [Na^+^]_i_ are rarely tracked in conductance-based models (important exceptions in [42–45]), although in thin active processes, such as axons and dendrites, they may substantially affect neuronal activity. Indeed, the effects of [Na^+^]_i_ on a variety of Ca^2+^-dependent processes were previously demonstrated: reducing Na^+^ extrusion or inhibiting the Na^+^-Ca^2+^ exchanger were shown to extend [Ca^2+^]_i_ transients, and thus to facilitate Ca^2+^-dependent mechanisms, such as synaptic plasticity and learning [29,46,47].

The activity-dependent Na^+^-K^+^ pump-mediated outward current is an important factor protecting neurons from a runaway positive feedback loop (S8B and S8C Fig). Without this current, the high [Ca^2+^]_i_ during and immediately after stimulation would result in a strong inward current (as demonstrated using ouabain—Fig 2G, red line) that would in turn evoke high frequency spiking, and thus a further increase in [Ca^2+^]_i_.

### Relevance to Other Brain Areas

Since the mechanism described here contains mostly elements which are ubiquitous in neurons, we argue that it is relevant (wholly or partially) to other brain areas. The essential non-trivial building blocks required to produce persistent activity by this mechanism are: a) changes in [Na^+^]_i_ and [Ca^2+^]_i_ in thin processes (axon or dendrites), attained by (back)-propagation of action potentials and/or excitatory synaptic activity; b) Co-localization of an excitatory Ca^2+^-dependent conductance at the site of [Na^+^]_i_ changes; c) Low density of [Na^+^]_i_ active extrusion mechanisms at the site of [Na^+^]_i_ changes.

Thus, the proposed mechanism is very likely to explain persistent activity in other brain areas, most likely with some variation of the time scale and of the factors necessary to evoke it. For example, [Na^+^]_i_ dynamics may take alternative forms in other neuronal types, as a function of the spatial distribution of Na^+^ channels and Na^+^-K^+^ pumps as well as cell morphology. Moreover, the persistent activity may be either superthreshold, i.e. persistent firing, or long term integrative changes in membrane potential and excitability. It may be assumed that very slow changes in resting potential or firing rate, such as those produced by the mechanism presented here, may be under-reported because they are frequently filtered out and require prolonged recording sessions.

Furthermore, the importance of the mechanism proposed by us is beyond the scope of persistent activity. We show that the long-term behavior of intracellular Ca^2+^ depends upon past activity—an idea previously presented in [29], though in a much shorter time scale. Given that Ca^2+^ is an important cellular signaling molecule, long-term changes in its concentration may broaden the time scale of processes such as synaptic plasticity [47], activity-dependent gene expression, and more. Similarly to membrane potential changes, reports about [Ca^2+^]_i_ changes may also suffer from "time-scale bias," wherein long-term changes in [Ca^2+^]_i_ seems to be regularly filtered out or not recorded due to photo damage and dye-bleaching concerns.

An even broader consequence is the ability of Na^+^ to integrate activity (discussed above), which at the very least causes changes to Na^+^ reversal potential. Thus, persistent activity is only one of several end results of the mechanism we describe. Our results at least demonstrate that changes in [Na^+^]_i_ should not be regarded as negligible in conceptual and computational models of neuronal activity.

## Materials and Methods

### Animals

C57BL/6J and BalbC male mice were maintained in the SPF mouse facility of the Hebrew University of Jerusalem under veterinary supervision, according to National Institutes of Health standards, with food and water ad libitum and lights on from 7:00 A.M. to 7:00 P.M. Eight- to twenty-week-old mice (25–35 g) were held in groups of 5–10 mice per cage. All experiments were approved by the Animal Care and Use Committee of the Hebrew University (permit number: NS-12-13310-4). Mice were anesthetized for in vivo experiments (ketamine, medatomidine). For in vitro experiments, mice were anesthetized (pentobarbitone) and killed by cervical dislocation.

### Natural Secretion Collection

Secretions for in vivo recordings were collected from C57BL/6J and BalbC females (housed in the animal facility of the Hebrew University). Samples were pooled and immediately frozen in liquid nitrogen and stored at −80°C until use. For urine collection, mice were gently held over a plastic sheet until they urinated. Vaginal secretions were collected by flushing the vagina with 30μl of ringer’s solution repeatedly. 20 μl were stored. For saliva collection, isoproterenol hydrochloride (0.2 mg/100 g) and pilocarpine (0.05 mg/100 g) were injected i.p. to increase salivation [48]. Following a delay of 5 min, saliva was collected from the mouth using a micropipette. Stimuli were diluted in Ringer’s solution.

### In Vivo Electrophysiology

In vivo multi-unit activity followed by electrical stimuli was recorded in anesthetized mice (ketamine, 10 mg/kg, medatomidine, Pfizer, 1 mg/kg). A recording electrode (glass micropipette filled with 1 M potassium acetate) was placed in the AOB external plexiform layer using a micromanipulator (Luigs and Neumann). A stimulating coaxial bipolar electrode was inserted through the medial frontal lobe, to the point where contact was made with the vomeronasal nerve and field potentials appeared in the AOB in response to brief stimuli. A train of brief shocks (0.1 ms, 1–100 V), given at 2 Hz for 2.5 s was applied to the vomeronasal nerve at intervals of 60 s via an isolated stimulator.

Electrophysiological recordings of AOB neurons followed by natural stimuli were performed as previously described in detail [18]. Briefly, BalbC mice were anesthetized with 100 mg/kg ketamine and 10 mg/kg xylazine. A tracheotomy was made using a polyethylene tube to allow breathing during flushing; a cuff electrode was placed on the sympathetic nerve trunk with the carotid serving as a scaffold. Incisions were closed and the mouse was placed in a custom-built stereotaxic apparatus where anesthesia was maintained throughout the entire experiment with 0.5–1% isoflurane in O_2_. A craniotomy was opened immediately rostral to the rhinal sinus, the dura was removed around the penetration site, and electrophysiological probes were advanced into the AOB using an electronic micromanipulator (MP-285; Sutter instruments). All recordings were made with 32 channel probes (NeuroNexus Technologies). During each trial, 2 μl of stimulus solution was placed directly in the nostril (“stimulus application”) and after 20 s, a square-wave stimulation train (duration: 1.6 s, current: ±120 μA, frequency: 30 Hz) was applied through the sympathetic nerve cuff electrode to induce VNO pumping and, accordingly, stimulus entry to the VNO lumen (“sympathetic stimulation”). A pump was turned on 40 s after each stimulus presentation, followed (after 10 s) by application of Ringer’s solution (1–2 ml) to the nostril that was flushed through the nasopalatine duct to cleanse the nasal cavity. 20 s after Ringer's application sympathetic stimulation was performed to ensure the VNO lumen cleansing (the second stimulation).

Using an RZ2 processor, PZ2 preamplifier, and two RA16CH head-stage amplifiers (Tucker-Davis Technologies), neuronal activity was sampled at 25 kHz and band-pass filtered at 0.3–5 kHz. Custom MATLAB (Mathworks) programs were used to extract spike waveforms. Spikes were sorted automatically according to their projections on two principle components using KlustaKwik [49] and then manually verified and adjusted using the Klusters program [50].

### Slice Preparation

Mice were anesthetized (pentobarbitone; 60 mg/kg) and killed by cervical dislocation. Olfactory bulbs were dissected into a physiological solution containing the following (mM): 125 NaCl, 25 NaHCO_3_, 5 glucose, 3 KCl, 2 CaCl_2_, 1.3 NaH_2_PO_4_, and 1 MgCl_2_, oxygenated by bubbling through a 95% O_2_ and 5% CO_2_ mixture, pH 7.4, 36°C. Parasagittal olfactory bulb slices, 300–400 μm thick, were prepared and equilibrated for 0.5–3h in the same solution at physiological temperature [51].

For electrophysiological recordings, slices were submerged in oxygenated physiological solution (identical to above) at room temperature in a recording chamber and perfused at a constant rate of 5–7 ml/min. To test the effect of substituting Na^+^ by Li^+^, equimolar amount of LiCl was used instead of NaCl. To test the effect of Ca^2+^ removal, equimolar amount of MgCl_2_ was used instead of CaCl_2_. Where indicated, picrotoxin (100 μM) was added to the bath solution to block GABA_A_ receptors, or ouabain (Tocris Bioscience) was added to the bath solution in excess (10–100 μM) to block the Na^+^-K^+^ pump.

### Slice Electrophysiology

For electrophysiological recordings, we used an Olympus BX61WIF microscope equipped with a motorized stage and manipulators (Luigs and Neumann), pulse generator (Master8, A.M.P.I.), isolated stimulator (ISOFlex, A.M.P.I.), and a MultiClamp 700B amplifier (Molecular Devices).

Mitral cells were visualized using infrared differential interference contrast (DIC) video microscopy via a 40x or a 60x water-immersion objective. Mitral neurons were identified by the location of the cell body on the ventral side of the external plexiform layer of the AOB. Whole-cell recordings were performed using borosilicate pipettes filled with standard intracellular recording solution containing the following (mM): 130 K-gluconate, 10 Na-gluconate, 10 HEPES, 10 phosphocreatine, 4 MgATP, 0.3 NaGTP, and 4 NaCl (pH = 7.25 with KOH, 5–12 MΩ). When BAPTA was used, BAPTA—tetrapotassium (Sigma) was dissolved in this solution to a final concentration of 5 mM. Seal resistance was at least 2 GΩ and typically 5–10 GΩ.

In most experiments, a 4-s-long spike train was evoked by injecting a series of depolarizing pulses (rate, 1–30 Hz; amplitude, 1–2 nA; pulse duration, 10 ms). In the hybrid-clamp procedure, membrane potential was clamped to −80 or −70mV throughout the experiment, excluding 4 s periods during which the amplifier was switched to current-clamp mode to deliver the train of current pulses. It should be noted that the hybrid-clamp methodology has been proven useful for investigating the firing activity of neurons, since by preventing most of the feedback and interference that ongoing firing activity may elicit upon itself, it enables a relatively clean examination of the underlying currents.

All amplified signals were digitized at 2–20 kHz using a National Instruments board and homemade software written in LabVIEW (National Instruments).

A unitary measurement of EPSC was done by filling a cell with Alexa 488 (Life Technologies) and visually positioning a bipolar theta electrode filled with physiological solution and Alexa 488 close to one of the dendritic tufts (Fig 8C).

### Calcium Imaging

For calcium imaging experiments, Oregon Green BAPTA-1 (OGB-1, Life Technologies, 50 μM) was added to the pipette solution. Fluorescence signals were recorded during the hybrid clamp protocol using a high speed camera (MiCAM Ultima, Brainvisions) and converted to (*F−F*_*min*_)/*F*_*min*_ratio after subtracting the ongoing background signal. In order to perform imaging of the dendritic tuft, a 60x water-immersion objective was used, and the dye was allowed to fill the cell for >20 min before recording was started. Evoked spikes were used to increase fluorescence and facilitate the visual search for a dendritic tuft.

### Sodium Imaging

For sodium imaging experiments, SBFI salt (TEFLabs, 2mM) and Sodium Green salt (Life Technologies, 500uM) was added to a modified pipette solution containing (mM): 130 K-gluconate, 10 HEPES, 5 phosphocreatine, 4 MgATP, 0.3 NaGTP, 20 KCl, and 0.2 EGTA (pH = 7.2 with KOH, 5–12 MΩ). Fluorescence signals were recorded from the apical dendrite as in the case of calcium imaging. A standard Fura-2 filter set (Ex. 380 nm; Em. 510 nm, Chroma Technology) was used for SBFI imaging. In order to cancel the substantial dye bleaching, trials without stimulus were subtracted from stimulus trials.

### Data Analysis

Unless otherwise noted, recorded current or voltage traces were averaged for each recorded cell, and the presented result is the mean of the cell population.

Value error range reported is SEM unless otherwise noted.

For stimulus-induced in vivo AOB recordings, units from various sets of experiments were considered. Six hundred and sixty-three single units for which at least one stimulus was presented at least five times were considered. The procedure for identifying persistent responses was as follows: For each single trace (one unit, one presentation of one stimulus), the responses in consecutive 2 s bins were defined as significant if the rate within it was larger than the mean baseline rate by at least five times the SEM of the baseline rate. The bins spanned a period of 160 s, which includes the baseline period, stimulus delivery, and the VNO flushing period, extending 20 s after the sympathetic stimulation during flushing (second stimulation, see above). The response duration was defined as a period beginning and ending with a significant bin, and in which at least 85% of the bins were significant. If this response lasted more than 10 s following the second sympathetic stimulation, it was designated as a persistent response. Finally, units for which at least half of the responses were persistent for a given stimulus were defined as persistent.

The seven persistent firing units came from three sets of experiments differing in stimulus sets used. For three units the stimuli were undiluted saliva, urine, and vaginal secretions. In three other units the stimuli were saliva, 100F diluted urine, and vaginal secretions. For the remaining units, the stimuli were urine at 1F, 10F, and 30F dilutions.

### Computational Models

The abstract dynamical model was constructed in MATLAB/SIMULINK (Mathworks). See S1 Text for details and equations.

We constructed the conductance-based model using the NEURON simulation environment with Python [52,53]. The model was based on experimental measurements and morphological reconstruction of an AOB mitral cell [54], and included influx, diffusion, and extrusion of Na^+^ and Ca^2+^. It assumed a presence of active Na^+^ channels in the apical dendrites and tufts [24], as well as non-uniform channel properties across different compartments (Fig 4E, [55]). Evolutionary multi objective optimization algorithm [25,26] was used to find the model parameters, based on recorded electrophysiological and imaging data. Some membranal mechanisms were based upon published models hosted by ModelDB [56–60]. See S1 Text for additional information. The model code is available online at:

https://senselab.med.yale.edu/ModelDB/ShowModel.cshtml?model=185332

### Network Model

In order to test the response of the model mitral cell to natural stimuli, a simple two-layer network model was constructed. The firing response of the VNO sensory neurons was modeled using a simple ligand-receptor interaction [33] that triggers a semi-random spike train (Fig 8A). Thirteen of such sensory neurons converged on each of the mitral cell's dendritic tufts (Fig 8B) [35], where the synaptic current was modeled using a double exponential fit to the unitary response measured experimentally using a theta electrode (a bipolar micropipette pulled from a tubing with a θ-like cross-section) positioned next to a dye-filled mitral cell's dendritic tuft (Fig 8C).

## Supporting Information

S1 Text Describing the computational models construction steps and equations. (DOCX)

S1 Fig AOB mitral cells are capable of responding with persistent firing to natural stimuli in vivo. (A) Top: raster plots showing a single unit response to 10 repetitions of VNO stimulation by female saliva (red), urine (green), and vaginal secretion (blue). Bottom: PSTH of the changes in firing frequency. Shaded area denotes SEM. Vertical black lines denote the times of application of the solutions to the nasal cavity and the activation of the sympathetic nerve. (B) Mean change in firing frequency, measured during 20 s following stimulus flush (highlighted interval in a) for the same unit and stimuli as in (A). Error bars denote SEM.
(TIF)

S2 Fig Ca^2+^ buffering unmasks the outward current that follows a spike train. (A) The mean current following an evoked 4 s long spike train at 30 Hz in control conditions (blue) and in a mitral cell filled with 5mM BAPTA (green). Compare to Fig 2E.
(TIF)

S3 Fig The dynamics of the prolonged inward current is unrelated to the Ca^2+^ concentration in the soma. (A) Fluorescence signal recorded from the soma during repeated stimulation by spike trains (4 s long at 10, 15, 20,and 25 Hz, red bars). Inset: fluorescence microscope image of the mitral cell filled with OGB-1, showing the somatic area from which the signals were recorded (red circle). The fluorescence level recorded before the first train (dashed line) was used to calculate dF/F values. (B) Current traces recorded simultaneously along with the fluorescence signals shown in (A). (C)–(D) Two examples of scatter plots of the recorded current versus the simultaneously recorded somatic fluorescent signal. The data of the first 7 s following each spike train were discarded. (E)–(F) Same as (C)–(D), showing data from two cells with tuft fluorescence measurements. Sigmoid curves were fitted to the data (dashed lines).
(TIF)

S4 Fig Simplification of the reconstructed mitral cell morphology. (A) The detailed reconstruction of the mitral cells used for the model. Functional compartments are color coded [61]. (B) The simplified geometry of the mitral cell, where the functional compartments were reduced to cylindrical sections while preserving their membrane surface area and passive properties.
(TIF)

S5 Fig The spike amplitude modulation in the model is abolished when the model is modified such that current through voltage-gated Na^+^ channels does not result in changes in [Na^+^]_i_. (A)–(D) Firing responses of the real cell (left) and the modified model (right) to step current injection of 100, 150, 200, and 350 pA, respectively. Note the lack of spike amplitude modulation in the simulated traces (compare to Fig 4F–4I).
(TIF)

S6 Fig The fit of the computational model to the data is robust to moderate change in model parameters. (A) The distribution of the maximal inward current following 4 s of 30 Hz stimulation in a population of model neurons based on randomized parameter set, where each parameter is drawn from a uniform distribution spanning −10% through +10% relative to the original value (*n* = 1,200). (B) The distribution of the residual current 1 min post-stimulus, in the same population as in (A). (C) The bounds (red lines) containing 80% of the distribution of current traces calculated in the random neuron population (based on a Z score margins of 1.28, assuming normal distribution). Blue line: current trace calculated for the original set of parameters. Black line: Experimental data used in the evolutionary algorithm to find the model parameters.
(TIF)

S7 Fig Na^+^ imaging results fit the time course predicted by the computational model. (A) The fluorescence change (dF/F) of the Na^+^ indicator SBFI in the apical dendrite of five cells in control conditions (gray lines) and two cells in the presence of TTX (light blue lines) while a 30 Hz train stimulus was applied (black bar). Thick lines denote multi-cell average. Based on the same data as Fig 6F. (B) same as A, using the Na^+^ indicator Sodium Green in four cells in control conditions. (C) The Z score (based on pre-stimulus standard deviation) of the Na^+^ indicator Sodium Green fluorescence signal in the apical dendrite of four cells in control conditions (gray lines) while a 30 Hz train stimulus was applied (black bar). Upward direction denotes decreased fluorescence. Black thick line denotes multi-cell average. The red line denotes the tuft [Na^+^]_i_ predicted by the model, scaled in the Y direction.
(TIF)

S8 Fig An inward current slowly reacting to changes in [Ca^2+^]_i_ or exclusion of the Na^+^-K^+^ pump-mediated outward current cause a runaway positive feedback loop. (A) The response of an alternative model, which incorporates a Ca^2+^-dependent inward current with very slow kinetics, to a 4-s-long 50 pA current injection (red bar). (B) The model cell response to a 4-s-long 1 Hz spike train (red bar). (C) The response to the same stimulus as in (B) of an altered model, in which the Na^+^-K^+^ pump-mediated outward current was blocked.
(TIF)

## Abbreviations

AIS: axon initial segment
AOB: accessory olfactory bulb
CAN: calcium-activated non-selective
EPSC: excitatory post-synaptic current
PSTH: peristimulus time histogram
SBFI: sodium-binding benzofuran isophthalate
TTX: tetrodotoxin
VNO: vomeronasal organ
VSN: vomeronasal sensory neuron
