# Supplementary material for: Prolonged Intracellular Na+ Dynamics Govern Electrical Activity in Accessory Olfactory Bulb Mitral Cells
Source: PLoS Biol. 2015 Dec 16;13(12):e1002319. doi: 10.1371/journal.pbio.1002319 (PMC4684409; doi:10.1371/journal.pbio.1002319)
Supplement: S1 Text — (DOCX) [file pbio.1002319.s001.docx]

**Abstract model equations**

The abstract model contains the core elements hypothesized to be involved in generating prolonged currents in AOB mitral cells. It is a two dimensional dynamical model described by the following equations.

[Na^+^]_i_ and [Ca^2+^]_i_ dynamical variables increase according to the "voltage" (v) when v is above threshold (10). At rest, [Na^+^]_i_ decays to zero, while [Ca^2+^]_i_ decays to a level determined by [Na^+^]_i_:

$$\frac{d\left[Ca^{2+}\right]_{i}}{dt}=\left(v-10\right)H\left(v-10\right)+3\left[Na^{+}\right]_{i}-\left[Ca^{2+}\right]_{i}$$

v is a sum of external stimulation, an inward current that has a sigmoidal relationship with [Ca^2+^]_i_ and outward outward "pump" current that has a saturating relationship with [Ca^2+^]_i_:

$$v=I_{ext}+I_{CAN}-I_{pump}$$

$$I_{CAN}=1.5\left(1-\frac{1}{1+e^{\left[Ca^{2+}\right]_{i}-3}}\right)$$

$$I_{pump}=1-e^{\frac{-\left[Na^{+}\right]_{i}}{5}}$$

The Symulink model that implements these equations is available online along with the article data at: http://dx.doi.org/10.12751/g-node.vd9c21

**Detailed computational Model Construction**

The model code is available online at:

<https://senselab.med.yale.edu/ModelDB/ShowModel.cshtml?model=185332>

The following steps were followed in the construction of the computational model:

1. *Geometry and reference data*

In order to obtain reference (training) data, an AOB mitral cell was filled with 150 µM Alexa 488 while its response to a series of current pulses (in pA: –60, –30, 30, 60, 100, 150, 200 and 350) was recorded. The slice was later transferred to a two-photon microscope (Sutter Instrument MOM), where a Z stack of the neuron was taken using a Nikon 16x/0.80w objective with a 0.5 µM Z-step. The 3D morphology of the neuron was reconstructed semi-manually using Neuromantic open source software [1] (Fig 4E). The reference data for the slow processes was taken from another mitral cell that was filled with OGB-1 and stimulated using the hybrid clamp protocol at 1 Hz, 15 Hz and 30 Hz, while fluorescence signal was collected from one of its dendritic tufts.

1. *Geometry lumping*

The cell morphology was used to create a passive neuron model in the NEURON simulation environment with Python[2,3], which was divided into nine functional compartments (sections) – soma, axon hillock, axon initial segment, axon, basal dendrite, two apical dendrites and two dendritic tufts. The geometry was simplified by reducing the number of electrical compartments (segments) to only one per section (S4 Fig., B), using the method described in [4]. In brief, an evolutionary multi-objective optimization (EMOO) algorithm was used to find an axial resistance, diameter and length values for each cylindrical section (while preserving the membrane surface area of each section), that retain the following properties as closely as possible to the detailed cell morphology: a) attenuation of somatic voltage step as a function of distance from the soma; b) cell impedance and c) cell phase shift, both recorded at the soma, as a function of stimulation frequency. The EMOO framework code published by [4] was used, except for the sorting of individual cell within each front, that was done using a score based on normalization coefficients rather than on crowding distance. The results of this step are summarized in Table 1.

1. *Initial fast mechanisms properties fitting*

In order to reproduce the short-term firing properties of the neuron, the ionic mechanisms detailed below were introduced. The channels were selected from ModelDB [5], and their kinetics are usually derived from neocortical pyramidal cells (voltage shift and time constant factors were used to adjust them to AOB data). The simulation parameters were fitted using parallel EMOO on the ELSC computer cluster, where the objectives were: passive hyperpolarizing response, spike shape, I-F curve and spiking train envelope at 350pA current injection. The sum of squared errors between the data and the model outcome vectors was used as error measures. All basic units are mV, S/cm^2^ and msec. Underscore denotes a fixed simulation parameter that is set by the evolutionary optimization:

*General*

Dendritic factor ($\underline{DF}$) is used to account for reconstruction inaccuracies in the dendrites, such as omitted fine processes. It multiplies the membrane capacitance and passive conductance in the dendrites and axon (but not in the soma). Unless otherwise noted, standard Hodgkin-Huxley type gating variables were used:

$\dot{w}=\frac{w_{\infty}-w}{\tau_{w}}$ $\tau_{w}(t)=\frac{\underline{\tau_{w}^{*}}}{\alpha_{w}+\beta_{w}} w_{\infty}=\frac{\alpha_{w}}{\alpha_{w}+\beta_{w}}$

Where w is any gating variable, and $\underline{\tau_{w}^{*}}$is its time factor parameter.

*Na^+^ and K^+^ passive leak channels*

$I_{Na}^{leak}=g_{Na}^{leak}\left( V_{m}-E_{Na} \right)$, $I_{K}^{leak}=g_{K}^{leak}\left( V_{m}-\underline{E_{K}} \right)$

$$E_{Na}=\frac{RT}{F}ln\frac{\left[ {Na}^{+} \right]_{o}}{\left[ {Na}^{+} \right]_{i}}$$

$g_{tot}=\frac{\underline{DF}}{\underline{R_{m}}}$ in thin processes, $\frac{1}{\underline{R_{m}}}$ in the soma.

$g_{Na}^{leak}={g_{tot}}/\left( 1+\frac{\underline{E_{K}}-\underline{E_{leak}}}{\underline{E_{leak}}-\underline{E_{Na}}} \right)$ $g_{K}^{leak}=g_{tot}-g_{Na}^{leak}$

$\left[ {Na}^{+} \right]_{i}$ is initialized according to:$\left[ {Na}^{+} \right]_{i}^{*}=\left[ {Na}^{+} \right]_{o}e^{-\frac{\underline{E_{Na}}}{{RT}/F}}$ , but allowed to change during the simulation. The passive leak channels do not change $\left[ {Na}^{+} \right]_{i}$

*Transient voltage-gated Na^+^ channels*[6]

$$I_{Na}^{t}=\underline{g_{Na}^{t}}m^{3}h\left( V_{m}-E_{Na} \right)$$

$$\alpha_{m}=\frac{0.182\cdot\left( V_{m}+28 \right)}{1-e^{-\left( V_{m}+28 \right)/9}} \beta_{m}=\frac{-0.124\cdot\left( V_{m}+28 \right)}{1-e^{\left( V_{m}+28 \right)/9}}$$

$$\alpha_{h}=\frac{0.024\cdot\left( V_{m}+50 \right)}{1-e^{-\left( V_{m}+50 \right)/5}} \beta_{h}=\frac{-0.0091\cdot\left( V_{m}+75 \right)}{1-e^{\left( V_{m}+75 \right)/5}}h_{\infty}=\frac{1}{1+e^{\left( V_{m}+55 \right)/{6.2}}}$$

These channels were inserted to all of the sections except the axon and basal dendrite, including the apical dendrites [7] and their voltage response was shifted by $\underline{V_{shift}^{{Na}_{t}}}$, which is section-dependent[8].

*Fast and slow K^+^ channels*

These channels were inserted to all of the sections except the axon and basal dendrite and their voltage response was shifted globally by $\underline{V_{shift}^{K_{fs}}}$. Apical dendrites channel density is the intermediate value between the somatic and dendritic tuft densities.

$$I_{K}^{f}=\underline{g_{K}^{f}}n\left( V_{m}-\underline{E_{K}} \right)$$

$\alpha_{n}=\frac{0.02\cdot\left( V_{m}-25 \right)}{1-e^{-\left( V_{m}-25 \right)/9}} \beta_{n}=\frac{-0.002\cdot\left( V_{m}-25 \right)}{1-e^{\left( V_{m}-25 \right)/9}}$ [6]

$$I_{K}^{s}=\underline{g_{K}^{s}}a^{2}\frac{1}{2}(b+b^{*})\left( V_{m}-\underline{E_{K}} \right)$$

$$\alpha_{a}=\frac{0.0052\cdot\left( V_{m}-11.1 \right)}{1-e^{-\left( V_{m}-11 \right)/{13.1}}} \beta_{a}=0.01938\cdot e^{-\left( V_{m}+1.27 \right)/{71}}-0.0053$$

$$b_{\infty}=\frac{1}{1+e^{\left( V_{m}+58 \right)/{11}}} \tau_{b}=360+\left[ 1010+23.7\left( V_{m}+54 \right) \right]e^{-\left( \frac{V_{m}+75}{48} \right)^{2}}$$

$\tau_{b^{*}}=2350+1380\cdot e^{-0.01118V_{m}}-210\cdot e^{-0.0306V_{m}}$ $\dot{b^{*}}=\frac{b_{\infty}-b^{*}}{\tau_{b^{*}}}$ [9]

1. *Slow mechanisms properties fitting*

In order to reproduce the long-term slow phenomena (tuft fluorescence signal and prolonged current), the ionic mechanisms detailed below were introduced on top of the mechanisms responsible for the fast response. EMOO was again used for parameter fitting, using the recorded current and fluorescence with 15 Hz and 30 Hz stimulations as goals as well as the fluorescence at 1 Hz stimulation. A realistic voltage clamp modelling was used, taking into account the pipette resistance (6 MΩ) and the lack of space clamp.

*Transient voltage gated Ca^2+^ channel*

$$I_{Ca}^{t}=\underline{{-g}_{Ca}^{t}}m^{2}hh^{*}\cdot25\left[ 1-\frac{\underline{\left[ {Ca}^{2+} \right]_{i}}}{\left[ {Ca}^{2+} \right]_{o}}e^{\frac{V_{m}}{25}} \right]\frac{{V_{m}}/{25}}{e^{\frac{V_{m}}{25}}-1}$$

$$\alpha_{m}=\frac{0.1967\cdot\left( V_{m}-19.88 \right)}{1-e^{-\left( V_{m}-19.88 \right)/{10}}} \beta_{m}\left( t \right)={0.046\cdot e}^{{{-V}_{m}\left( t \right)}/{20.73}}$$

$$\alpha_{h}\left( t \right)={1.6\cdot{10}^{-4}\cdot e}^{{{-V}_{m}\left( t \right)}/{48.4}} \beta_{h}\left( t \right)=\frac{1}{1+e^{-\left( V_{m}\left( t \right)-39 \right)/{10}}}$$

$h^{*}=\frac{\underline{k_{i}}}{\underline{k_{i}}+\left[ {Ca}^{2+} \right]_{i}}$ [10]

The Ca^2+^ channels are inserted only at the dendritic tuft.

*Ca^2+^ and Na^+^ diffusion, extrusion and buffering (endogenous and exogenous)*

The Ca^2+^ mechanisms are only inserted in the dendritic tuft. Ca^2+^ diffuses radially between four concentric annuli according to a diffusion constant $\underline{{diff}_{{Ca}^{2+}}}$. Na^+^ diffuses both radially and longitudinally according to a diffusion constant $\underline{{diff}_{{Na}^{+}}}$. Na^+^ mechanisms are inserted to all sections.

Plasma membrane Ca^2+^ pump:

$$\left[ {Ca}^{2+} \right]_{i}+\left[ pump \right]\underset{\leftrightarrow}{1, \sqrt{\underline{\left[ {Ca}^{2+} \right]_{i}^{*}}}}\left[ pumpCa \right]$$

$[pumpCa]\underset{\leftrightarrow}{1, \sqrt{\underline{\left[ {Ca}^{2+} \right]_{i}^{*}}}}\left[ pump \right]$+$\left[ {Ca}^{2+} \right]_{o}$

$$\underline{\left[ PMCA \right]}=[pumpCa]+\left[ pump \right]$$

Buffering:

$$\left[ {Ca}^{2+} \right]_{i}+\left[ EndBuffer \right]\underset{\leftrightarrow}{100, 100\cdot\underline{k_{d}^{end}}}\left[ EndBufferCa \right]$$

$$\left[ {Ca}^{2+} \right]_{i}+\left[ ExBuffer \right]\underset{\leftrightarrow}{200,200\cdot\underline{k_{d}^{ex}}}\left[ ExBufferCa \right]$$

$$\underline{\left[ EndBufferTot \right]}=\left[ EndBufferCa \right]+\left[ EndBuffer \right]$$

The predicted fluorescence signal is calculated using the weighted average $\bar{\left[ ExBufferCa \right]}$ over the four annuli:

$$F=\left[ ExBufferTot \right]+\left( \underline{\mu_{f}}-1 \right)\overline{\left[ ExBufferCa \right]}$$

Na^+^-K^+^ pump:

$${3\left[ {Na}^{+} \right]}_{i}+\left[ pump \right]\underset{\leftrightarrow}{\underline{k_{1}},\underline{k_{2}}}\left[ pumpNa \right]$$

$[pumpNa]\underset{\leftrightarrow}{\underline{k_{3}},k_{4}}\left[ pump \right]$+${3\left[ {Na}^{+} \right]}_{o}$ $k_{4}=k_{1}k_{3}k_{2}^{-1}\left( \frac{\left[ {Na}^{+} \right]_{i}^{*}}{\left[ {Na}^{+} \right]_{o}} \right)^{3}$

$$\underline{\left[ NaKPump \right]}=[pumpNa]+\left[ pump \right]$$

Na^+^-Ca^2+^ exchanger:

$I_{NCX}=I_{NCX(max)}\frac{{\left[ {Na}^{+} \right]_{i}^{3}\left[ {Ca}^{2+} \right]_{o}e}^{\frac{\underline{\gamma}V_{m}F}{RT}}-{\left[ {Na}^{+} \right]_{o}^{3}\left[ {Ca}^{2+} \right]_{i}e}^{\frac{(\underline{\gamma}-1)V_{m}F}{RT}}}{\left( \underline{k_{m(Na)}^{3}}+\left[ {Na}^{+} \right]_{o}^{3} \right)\left( \underline{K_{m(Ca)}}+\left[ {Ca}^{2+} \right]_{o} \right)\left( 1+\underline{k_{sat}}e^{\frac{(\underline{\gamma}-1)V_{m}F}{RT}} \right)}$ [11]

I_CAN_ channels

$$I_{CAN}=\underline{g_{CAN}}m\left( V_{m}-E_{CAN} \right)$$

$$\alpha_{m}=\left( \frac{\left[ {Ca}^{2+} \right]_{i}}{\underline{{CAN}_{1/2}}} \right)^{\underline{\varphi}} \beta_{m}=1$$

After the parameters were fitted for the slow processes, the EMOO was run again to re-adjust the parameters for the fast responses. The final parameter values for all of the steps are summarized in Tables 1 and 2. The constrained parameters are summarized in Table 3.

**Table 1 –** Lumped geometry parameters of the final mitral cell model

|  | Diameter (µm) | Length (µm) | Axial resistance (Ω·cm) |
| --- | --- | --- | --- |
| Soma | 15.12 | 21.01 | 17.94 |
| Hillock | 3.25 | 9.47 | 58.84 |
| AIS | 2.91 | 23.75 |  |
| Axon | 2.08 | 294.28 |  |
| Basal dendrite (Basal) | 1.52 | 150.66 | 63.03 |
| Apical dendrite 1 (Apical 1) | 6.51 | 107.81 | 217.31 |
| Apical dendrite 2 (Apical 2) | 8.29 | 361.44 | 400.00 |
| Dendritic tuft 1 (Tuft 1) | 3.03 | 633.93 | 81.20 |
| Dendritic tuft 2 (Tuft2) | 1.12 | 500.00 | 44.11 |

**Table 2 –** Final parameter values of the mitral cell model following optimization.

| $\underline{DF}$ | 1.1 | | $\underline{\tau_{h,Ca}^{*}}$ | 17.57 | |
| --- | --- | --- | --- | --- | --- |
| $\underline{r_{m}} (\Omega\cdot{cm}^{2})$ | 6.912E4 | | $\underline{V_{shift}^{Ca}} (mV)$ | 10.29 | |
| $\underline{E_{K}} (mV)$ | –87.44 | | $\underline{k_{i}} (mM)$ | 11.66 | |
| $\underline{E_{Na}} (mV)$ | 59.25 | | $\underline{\left[ {Ca}^{2+} \right]_{i}^{*}} (nM)$ | 39.77 | |
| $\underline{E_{leak}} (mV)$ | –50.37 | | $\underline{\left[ PMCA \right]} (pmol/{cm}^{2})$ | Tuft 1 | 17.8 |
| $\underline{g_{Na}^{t}} (S/{{cm}^{2})}$ | Soma | 0.375 |  | Tuft 2 | 5.24 |
|  | Hillock | 0.454 | $\underline{\left[ NaKPump \right]} (pmol/{cm}^{2})$ | Soma | 40.47 |
|  | AIS | 1.941 |  | Hillock | 4.31 |
|  | Apical 1 | 0.001 |  | AIS | 6.72E-5 |
|  | Apical 2 | 0.016 |  | Axon | 0.5 |
|  | Tuft 1 | 0.014 |  | Apical, Tuft 1 | 3.47E-4 |
|  | Tuft 2 | 0.0005 |  | Apical, Tuft 2 | 1.29E-6 |
| $\underline{V_{shift}^{{Na}_{t}}} (mV)$ | Soma | 9.97 | $\underline{k_{d}^{end}}(\mu M)$ | 597.38 | |
|  | Hillock | 16.07 | $\underline{k_{d}^{ex}}(nM)$ | 237.95 | |
|  | AIS | 19.17 | $\underline{\left[ EndBufferTot \right]} (mM)$ | 10.13 | |
|  | Dendrites | 10.91 | $\underline{\mu_{f}}$ | 8.3 | |
| $\underline{\tau_{m,Na}^{*}}$ | 0.737 | | $\underline{k_{1}}(/[mM\cdot ms])$ | 1.841 | |
| $\underline{\tau_{h,Na}^{*}}$ | 1.223 | | $\underline{k_{2}}(/ms)$ | 0.022 | |
| $\underline{g_{K}^{f}}$ $(S/{{cm}^{2})}$ | Soma | 4.948E-6 | $\underline{k_{3}} (/[mM\cdot ms])$ | 2.313 | |
|  | Hillock | 0.003 | $I_{NCX(max)} ({mA}/{{cm}^{2})}$ | Tuft 1 | 16.485 |
|  | AIS | 0.022 |  | Tuft 2 | 17.933 |
|  | Tufts | 0.004 | $\underline{\gamma}$ | 0.292 | |
| $\underline{V_{shift}^{K_{f}}} (mV)$ | 6.91 | | $\underline{k_{m\left( Na \right)}} (mM)$ | 219.85 | |
| $\underline{\tau_{n,K(fast)}^{*}}$ | 0.118 | | $\underline{k_{m(Ca)}} (mM)$ | 0.333 | |
| $\underline{g_{K}^{s}} (S/{{cm}^{2})}$ | Soma | 5.256E-5 | $\underline{k_{sat}}$ | 0.078 | |
|  | Hillock | 0.295 | $\underline{g_{CAN}}(S/{{cm}^{2})}$ | Tuft 1 | 6.42E-6 |
|  | AIS | 0.168 |  | Tuft 2 | 7.26E-10 |
|  | Tufts | 0.004 | $\underline{{CAN}_{1/2}(nM)}$ | 93.05 | |
| $\underline{V_{shift}^{K_{s}}} (mV)$ | 42.62 | | $\underline{\varphi}$ | 5.09 | |
| $\underline{g_{Ca}^{t}} (S/{{cm}^{2})}$ | Tuft 1 | 0.024 | $\underline{{diff}_{{Ca}^{2+}} (\mu m/ms)}$ | 0.1 | |
|  | Tuft 2 | 0.007 | $\underline{{diff}_{{Na}^{+}}(\mu m/ms)}$ | 0.09 | |

**Table 3 –** Fixed (constrained) parameters of the mitral cell model.

| $C_{m}^{*} (\mu F/{cm}^{2})$ | 1 |
| --- | --- |
| $\left[ {Na}^{+} \right]_{o} (mM)$ | 151.3 |
| $\left[ {Ca}^{2+} \right]_{o} (mM)$ | 2.0 |
| $\left[ ExBufferTot \right]\equiv{[OGB]}_{i} (\mu M$) | 50 |
| $E_{CAN} (mV)$ | 15 |

**References**

1. Myatt DR, Hadlington T, Ascoli GA, Nasuto SJ. Neuromantic - from semi-manual to semi-automatic reconstruction of neuron morphology. Front Neuroinform. 2012;6: 4. doi:10.3389/fninf.2012.00004

2. Hines ML, Carnevale NT. The NEURON Simulation Environment. Neural Comput. MIT Press  238 Main St., Suite 500, Cambridge, MA 02142-1046 USA journals-info@mit.edu; 1997;9: 1179–1209. doi:10.1162/neco.1997.9.6.1179

3. Hines ML, Davison AP, Muller E. NEURON and Python. Front Neuroinform. 2009;3: 1. doi:10.3389/neuro.11.001.2009

4. Bahl A, Stemmler MB, Herz AVMM, Roth A. Automated optimization of a reduced layer 5 pyramidal cell model based on experimental data. J Neurosci Methods. 2012;210: 22–34. doi:10.1016/j.jneumeth.2012.04.006

5. Hines ML, Morse T, Migliore M, Carnevale NT, Shepherd GM. ModelDB: A Database to Support Computational Neuroscience. J Comput Neurosci. 2004;17: 7–11. doi:10.1023/B:JCNS.0000023869.22017.2e

6. Mainen ZF, Sejnowski TJ. Influence of dendritic structure on firing pattern in model neocortical neurons. Nature. 1996;382: 363–6. doi:10.1038/382363a0

7. Ma J, Lowe G. Action Potential Backpropagation and Multiglomerular Signaling in the Rat Vomeronasal System. J Neurosci. 2004;24: 9341–9352. doi:10.1523/JNEUROSCI.1782-04.2004

8. Colbert CM, Pan E. Ion channel properties underlying axonal action potential initiation in pyramidal neurons. Nat Neurosci. 2002;5: 533–538. doi:10.1038/nn0602-857

9. Korngreen A, Kaiser KMM, Zilberter Y. Subthreshold inactivation of voltage-gated K+ channels modulates action potentials in neocortical bitufted interneurones from rats. J Physiol. 2005;562: 421–37. doi:10.1113/jphysiol.2004.077032

10. Lazarewicz MT, Migliore M, Ascoli GA. A new bursting model of CA3 pyramidal cell physiology suggests multiple locations for spike initiation. Biosystems. 2002;67: 129–137. doi:10.1016/S0303-2647(02)00071-0

11. Courtemanche M, Ramirez RJ, Nattel S. Ionic mechanisms underlying human atrial action potential properties: insights from a mathematical model. Am J Physiol Hear Circ Physiol. 1998;275: H301–321. Available: http://ajpheart.physiology.org/content/275/1/H301.long
