# Supplementary figures and images for: Prolonged Intracellular Na+ Dynamics Govern Electrical Activity in Accessory Olfactory Bulb Mitral Cells
Source: PLoS Biol. 2015 Dec 16;13(12):e1002319. doi: 10.1371/journal.pbio.1002319 (PMC4684409; doi:10.1371/journal.pbio.1002319)

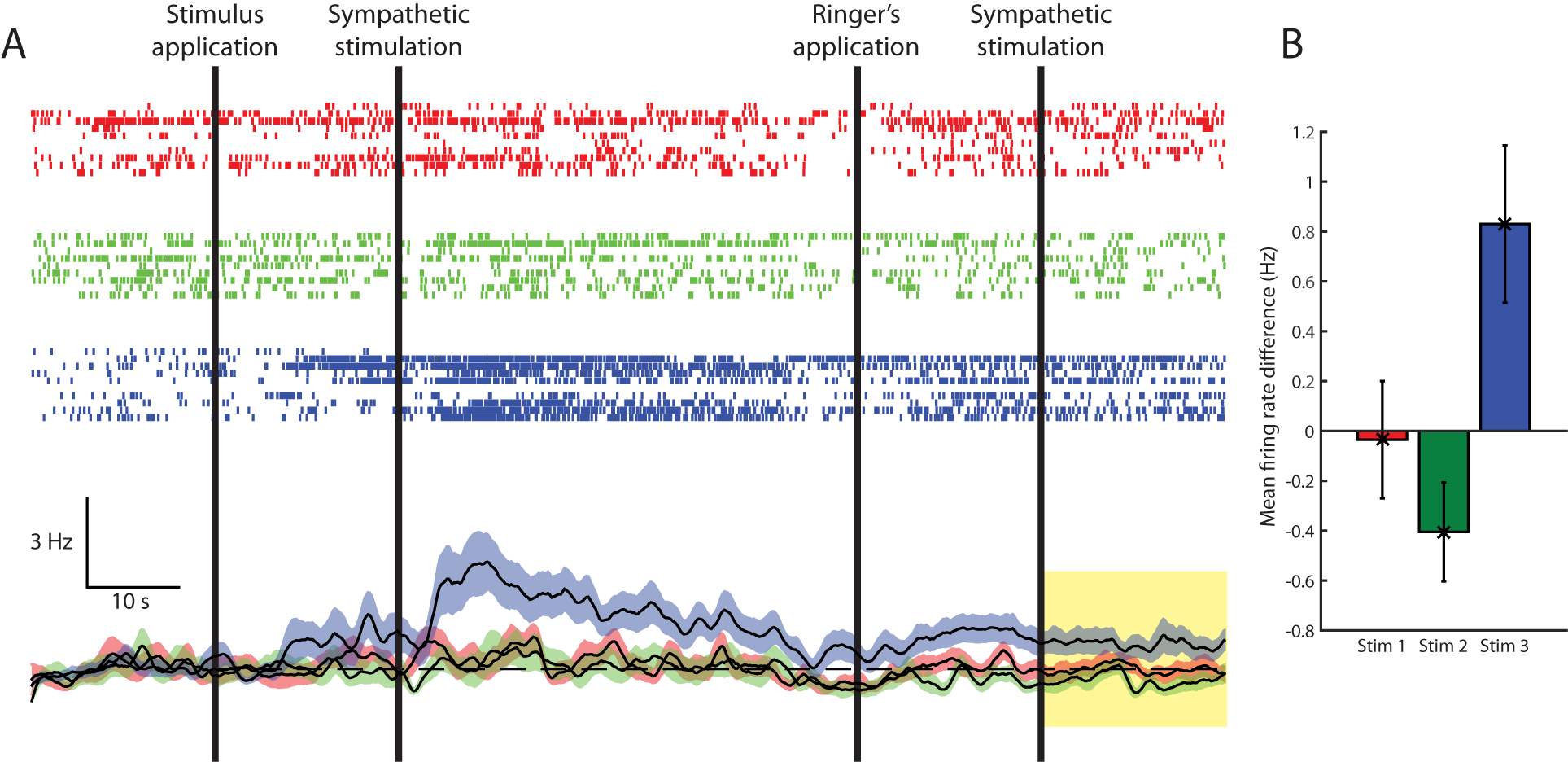

Supplement: S1 Fig — (A) Top: raster plots showing a single unit response to 10 repetitions of VNO stimulation by female saliva (red), urine (green), and vaginal secretion (blue). Bottom: PSTH of the changes in firing frequency. Shaded area denotes SEM. Vertical black lines denote the times of application of the solutions to the nasal cavity and the activation of the sympathetic nerve. (B) Mean change in firing frequency, measured during 20 s following stimulus flush (highlighted interval in a) for the same unit and stimuli as in (A). Error bars denote SEM. (TIF) [file pbio.1002319.s002.tif]

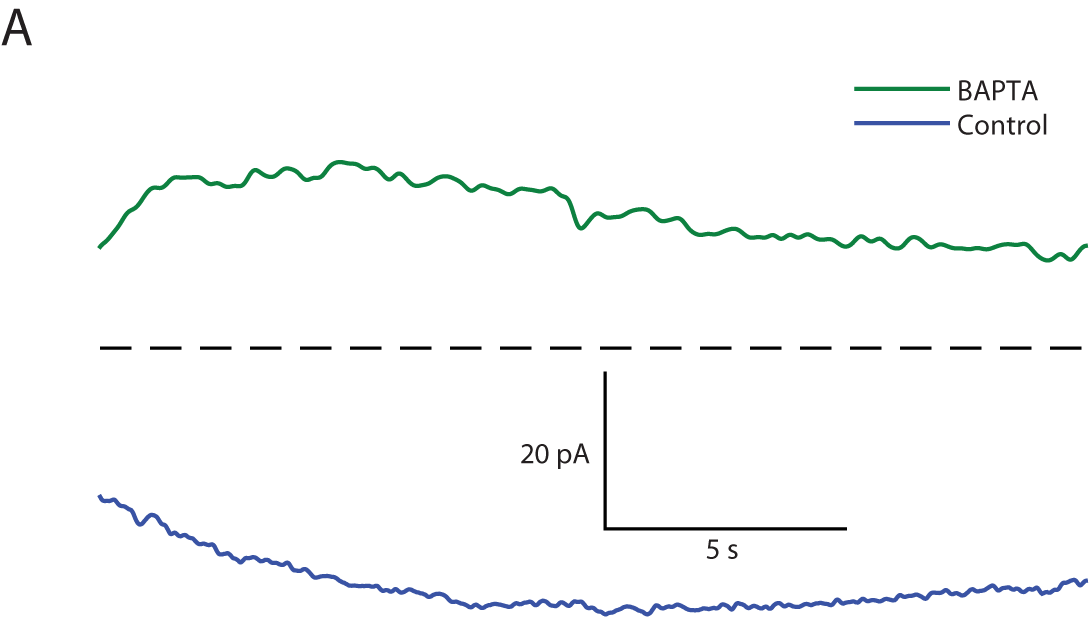

Supplement: S2 Fig — (A) The mean current following an evoked 4 s long spike train at 30 Hz in control conditions (blue) and in a mitral cell filled with 5mM BAPTA (green). Compare to Fig 2E. (TIF) [file pbio.1002319.s003.tif]

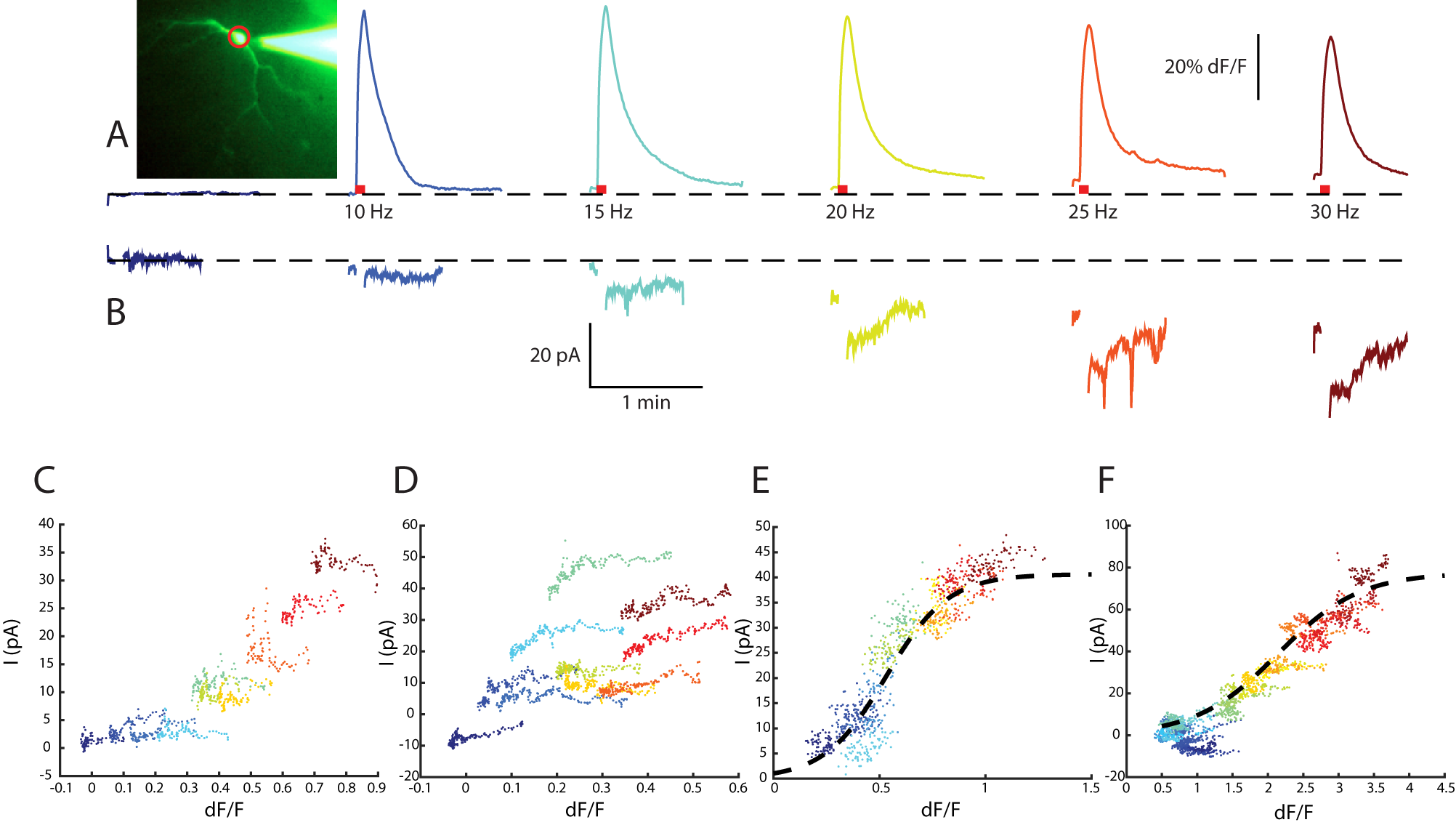

Supplement: S3 Fig — (A) Fluorescence signal recorded from the soma during repeated stimulation by spike trains (4 s long at 10, 15, 20,and 25 Hz, red bars). Inset: fluorescence microscope image of the mitral cell filled with OGB-1, showing the somatic area from which the signals were recorded (red circle). The fluorescence level recorded before the first train (dashed line) was used to calculate dF/F values. (B) Current traces recorded simultaneously along with the fluorescence signals shown in (A). (C)–(D) Two examples of scatter plots of the recorded current versus the simultaneously recorded somatic fluorescent signal. The data of the first 7 s following each spike train were discarded. (E)–(F) Same as (C)–(D), showing data from two cells with tuft fluorescence measurements. Sigmoid curves were fitted to the data (dashed lines). (TIF) [file pbio.1002319.s004.tif]

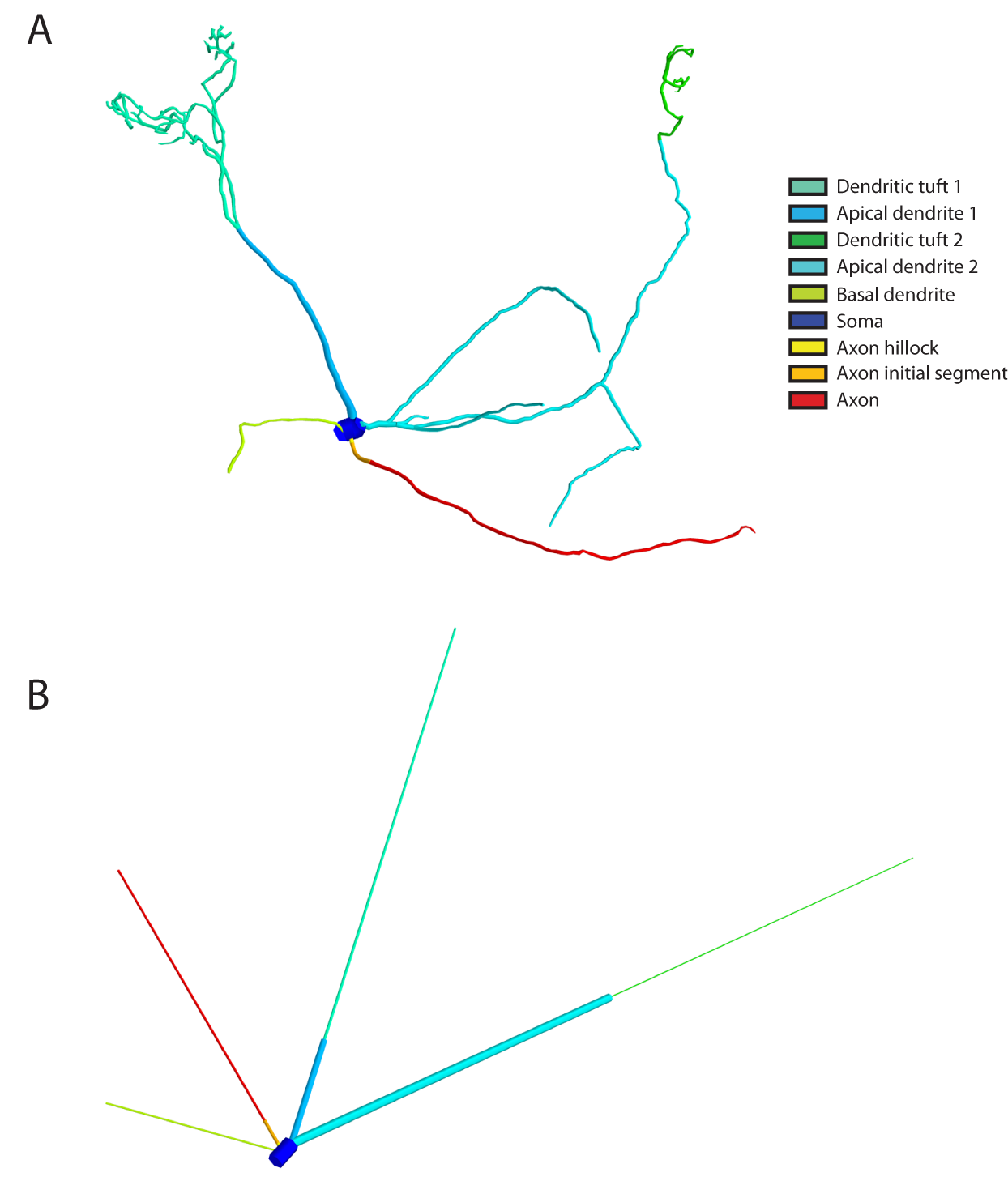

Supplement: S4 Fig — (A) The detailed reconstruction of the mitral cells used for the model. Functional compartments are color coded [61]. (B) The simplified geometry of the mitral cell, where the functional compartments were reduced to cylindrical sections while preserving their membrane surface area and passive properties. (TIF) [file pbio.1002319.s005.tif]

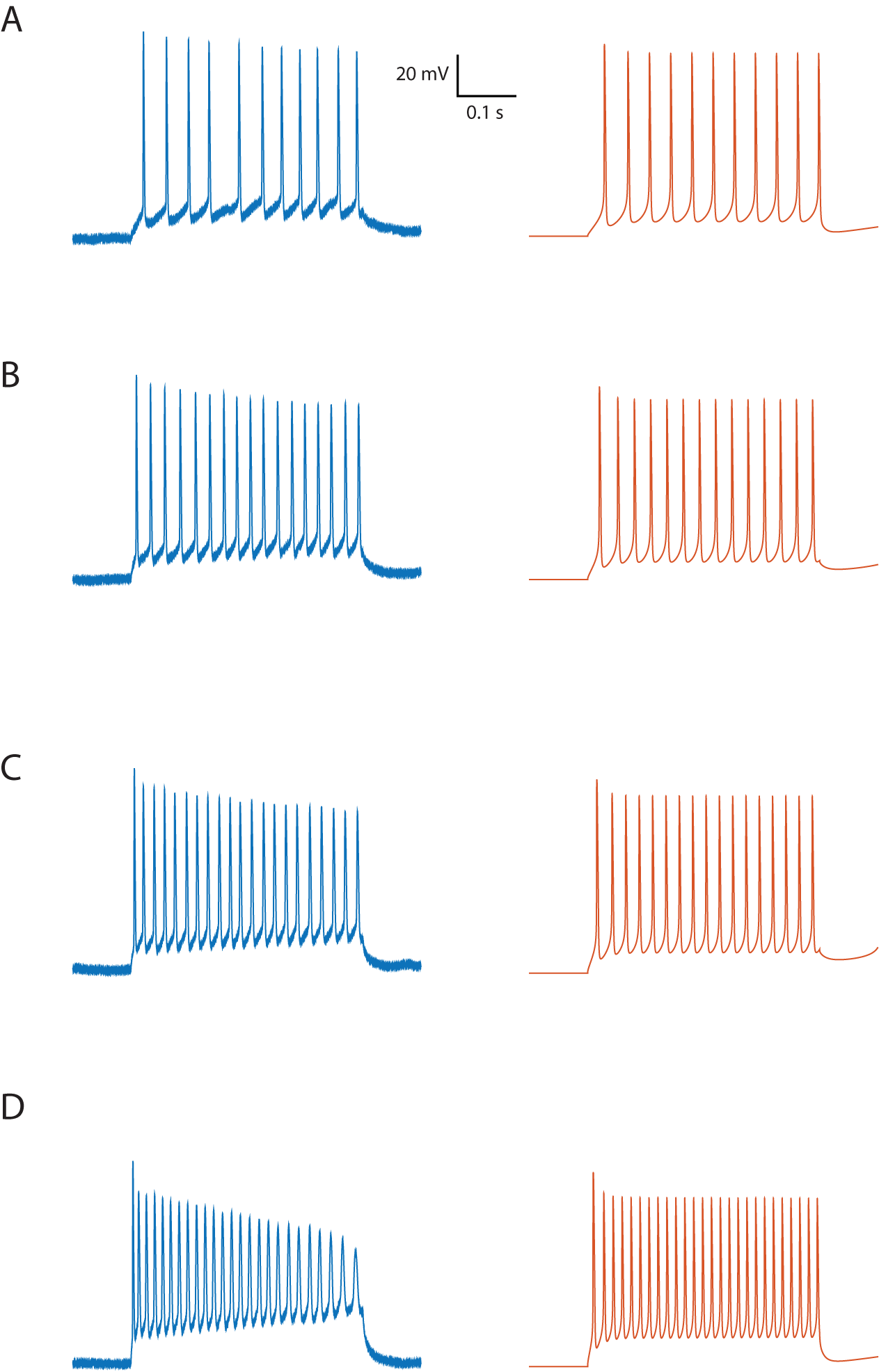

Supplement: S5 Fig — (A)–(D) Firing responses of the real cell (left) and the modified model (right) to step current injection of 100, 150, 200, and 350 pA, respectively. Note the lack of spike amplitude modulation in the simulated traces (compare to Fig 4F–4I). (TIF) [file pbio.1002319.s006.tif]

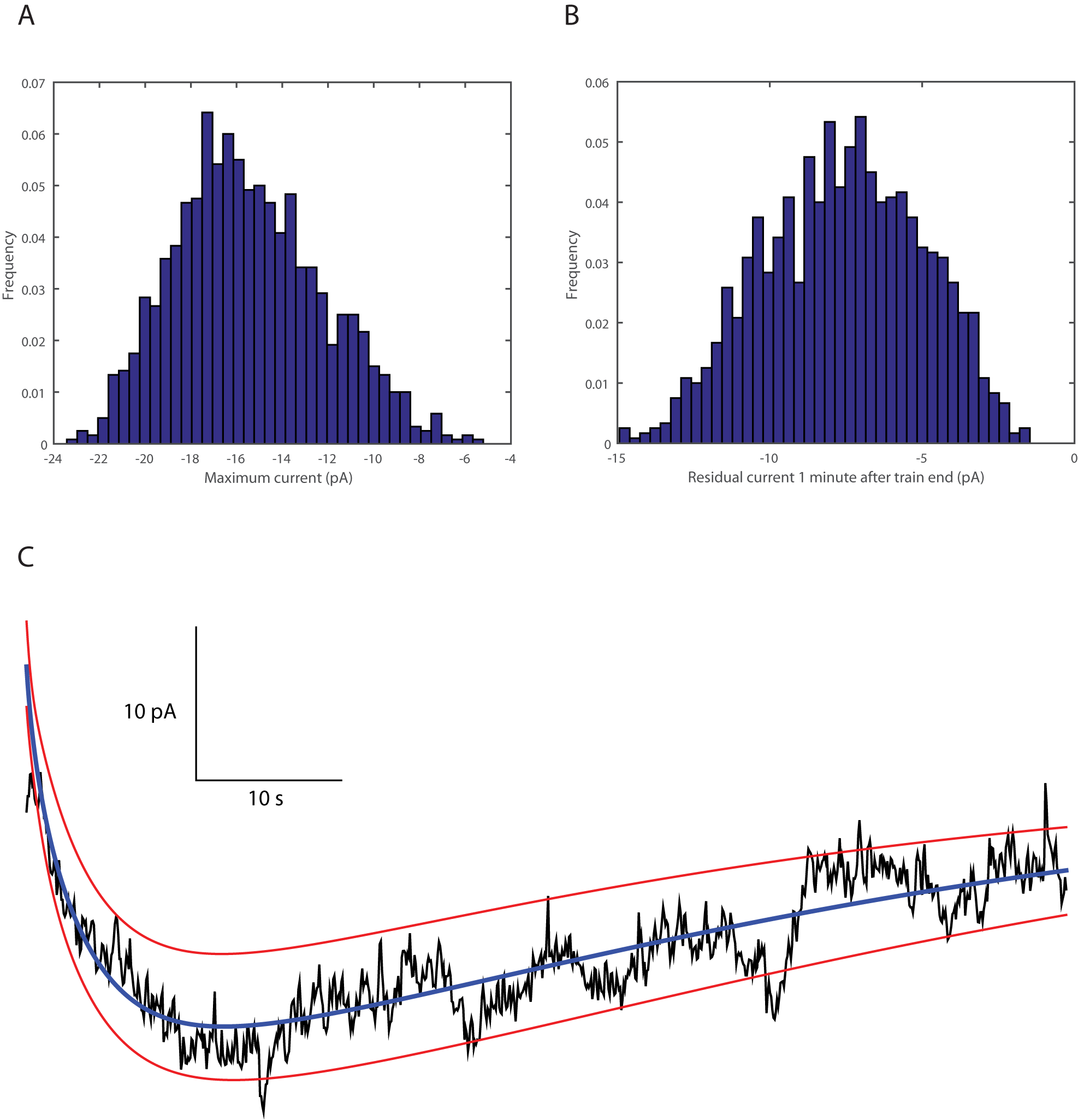

Supplement: S6 Fig — (A) The distribution of the maximal inward current following 4 s of 30 Hz stimulation in a population of model neurons based on randomized parameter set, where each parameter is drawn from a uniform distribution spanning −10% through +10% relative to the original value (n = 1,200). (B) The distribution of the residual current 1 min post-stimulus, in the same population as in (A). (C) The bounds (red lines) containing 80% of the distribution of current traces calculated in the random neuron population (based on a Z score margins of 1.28, assuming normal distribution). Blue line: current trace calculated for the original set of parameters. Black line: Experimental data used in the evolutionary algorithm to find the model parameters. (TIF) [file pbio.1002319.s007.tif]

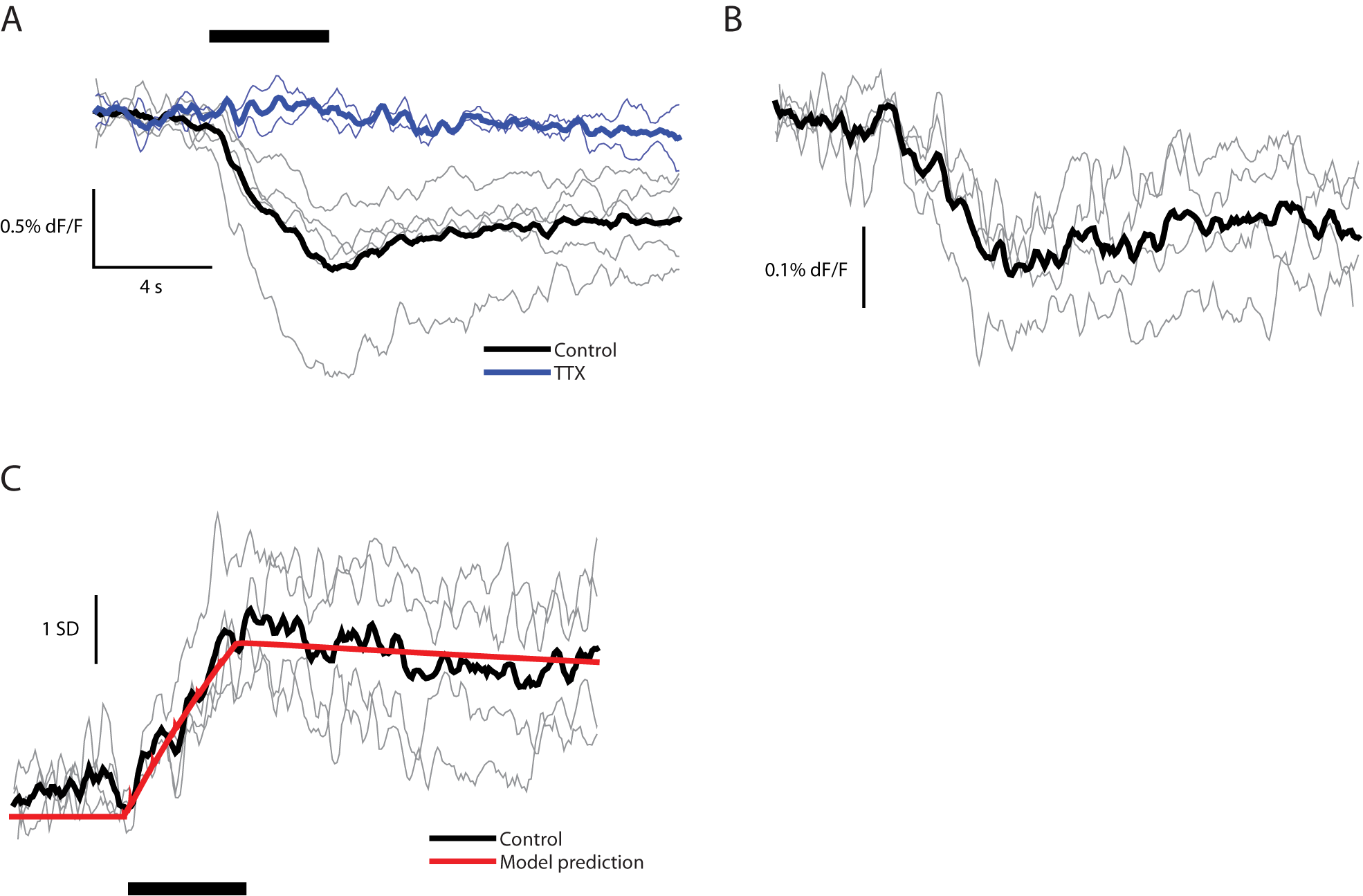

Supplement: S7 Fig — (A) The fluorescence change (dF/F) of the Na+ indicator SBFI in the apical dendrite of five cells in control conditions (gray lines) and two cells in the presence of TTX (light blue lines) while a 30 Hz train stimulus was applied (black bar). Thick lines denote multi-cell average. Based on the same data as Fig 6F. (B) same as A, using the Na+ indicator Sodium Green in four cells in control conditions. (C) The Z score (based on pre-stimulus standard deviation) of the Na+ indicator Sodium Green fluorescence signal in the apical dendrite of four cells in control conditions (gray lines) while a 30 Hz train stimulus was applied (black bar). Upward direction denotes decreased fluorescence. Black thick line denotes multi-cell average. The red line denotes the tuft [Na+]i predicted by the model, scaled in the Y direction. (TIF) [file pbio.1002319.s008.tif]

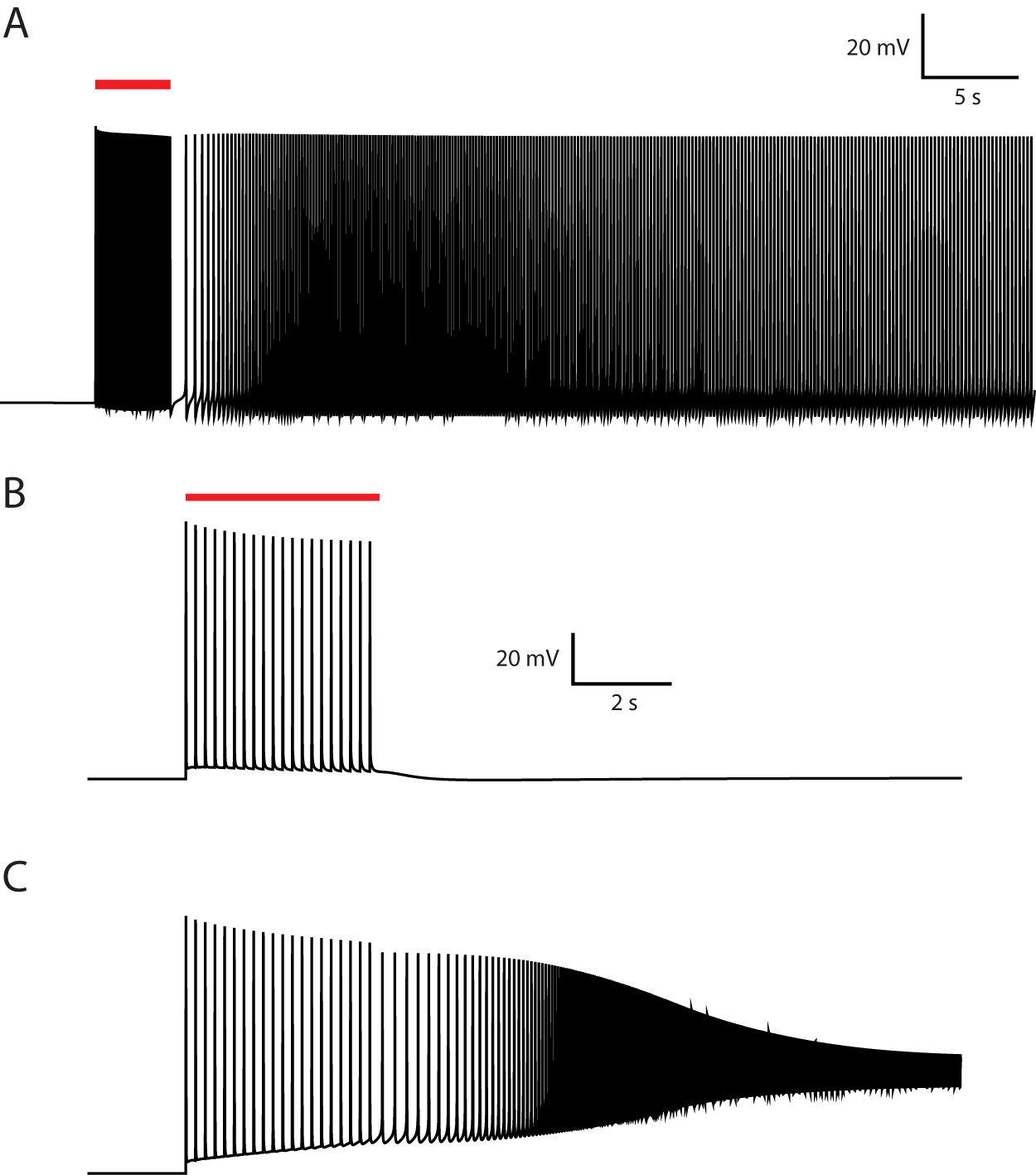

Supplement: S8 Fig — (A) The response of an alternative model, which incorporates a Ca2+-dependent inward current with very slow kinetics, to a 4-s-long 50 pA current injection (red bar). (B) The model cell response to a 4-s-long 1 Hz spike train (red bar). (C) The response to the same stimulus as in (B) of an altered model, in which the Na+-K+ pump-mediated outward current was blocked. (TIF) [file pbio.1002319.s009.tif]
